# Distinct Features of Cap Binding by eIF4E1b Proteins

**DOI:** 10.1016/j.jmb.2014.11.009

**Published:** 2015-01-30

**Authors:** Dorota Kubacka, Ricardo Núñez Miguel, Nicola Minshall, Edward Darzynkiewicz, Nancy Standart, Joanna Zuberek

**Affiliations:** 1 Division of Biophysics, Institute of Experimental Physics, Faculty of Physics, University of Warsaw, Warsaw 02-089, Poland; 2 Department of Biochemistry, University of Cambridge, Tennis Court Road, Cambridge CB2 1QW, UK; 3 Centre of New Technologies, University of Warsaw, Warsaw 02-089, Poland

**Keywords:** eukaryotic initiation factor, eIF4E1b, translational repression, binding affinity, *Xenopus laevis*

## Abstract

eIF4E1b, closely related to the canonical translation initiation factor 4E (eIF4E1a), cap-binding protein is highly expressed in mouse, *Xenopus* and zebrafish oocytes. We have previously characterized eIF4E1b as a component of the CPEB mRNP translation repressor complex along with the eIF4E-binding protein 4E-Transporter, the Xp54/DDX6 RNA helicase and additional RNA-binding proteins. eIF4E1b exhibited only very weak interactions with m^7^GTP-Sepharose and, rather than binding eIF4G, interacted with 4E-T. Here we undertook a detailed examination of both *Xenopus* and human eIF4E1b interactions with cap analogues using fluorescence titration and homology modeling. The predicted structure of eIF4E1b maintains the α + β fold characteristic of eIF4E proteins and its cap-binding pocket is similarly arranged by critical amino acids: Trp56, Trp102, Glu103, Trp166, Arg112, Arg157 and Lys162 and residues of the C-terminal loop. However, we demonstrate that eIF4E1b is 3-fold less well able to bind the cap than eIF4E1a, both proteins being highly stimulated by methylation at N^7^ of guanine. Moreover, eIF4E1b proteins are distinguishable from eIF4E1a by a set of conserved amino acid substitutions, several of which are located near to cap-binding residues. Indeed, eIF4E1b possesses several distinct features, namely, enhancement of cap binding by a benzyl group at N^7^ position of guanine, a reduced response to increasing length of the phosphate chain and increased binding to a cap separated by a linker from Sepharose, suggesting differences in the arrangement of the protein's core. In agreement, mutagenesis of the amino acids differentiating eIF4E1b from eIF4E1a reduces cap binding by eIF4E1a 2-fold, demonstrating their role in modulating cap binding.

## Highlights

• Sequence analysis of vertebrate eIF4E1a and eIF4E1b proteins identified a set of conserved substitutions, including those near to cap-binding residues.
• The fluorescence titration assay revealed that human and *Xenopus* eIF4E1b have 3-fold lower affinity for m^7^GTP than the eIF4E1a proteins.
• Additional distinct features of cap binding by eIF4E1b suggest differences in the arrangement of the protein's core and its C-terminal loop.
• Mutagenesis of the distinguishing amino acids reduced cap binding by eIF4E1a 2-fold, demonstrating their role in modulating affinity to m^7^GTP.

## Graphical abstract

**Figure:**
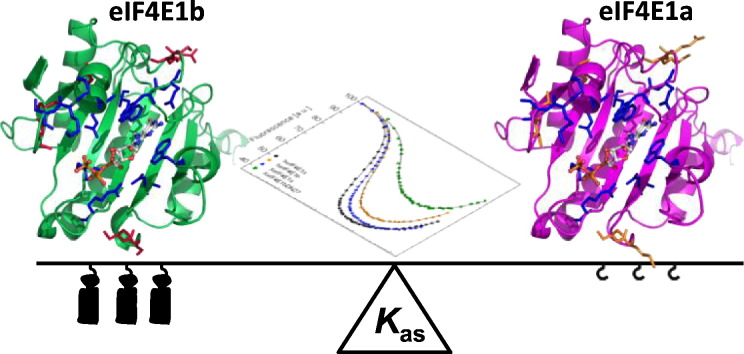

## Introduction

The cap-binding protein eIF4E is central to protein synthesis in eukaryotes. eIF4E is a small, approximately 25 kDa protein, whose structure resembles a cupped hand formed by an eight-stranded antiparallel β-sheet, with three α-helices on its convex side. The eIF4E cavity specifically interacts with the m^7^G(5′)ppp(5′)N cap (where N is typically G or A) added to RNA polymerase II transcripts. The indol rings of conserved tryptophans in eIF4E sandwich the N^7^-methyl guanine base via cation–π stacking interactions that are stabilized by van der Waals contacts and salt bridges with additional tryptophan and positively charged residues [1,2]. eIF4E is a component of the eIF4F complex, which also includes the large scaffold protein eIF4G and the eIF4A helicase and promotes translation initiation by mediating the interaction between mRNA and eIF4G that has additional factor binding sites including that for the multisubunit complex eIF3, thus enabling the recruitment of the small ribosomal subunit to the 5′ end of mRNA [3,4]. eIF4E is considered to be a proto-oncogene, since its overexpression causes tumorigenic transformation of fibroblasts, and high levels of eIF4E have also been implicated in autism-like behaviors [5–7].

Multiple eIF4E homologues have been identified in vertebrates, *Drosophila melanogaster*, *Caenorhabditis elegans*, *Leishmania major*, *Arabidopsis thaliana* and *Schizosaccharomyces pombe*, among others [8]. The initial classification of eIF4E-family members focused on their sequence similarity and conservation of the characteristic tryptophan residues that are located close by or form the active-site center (Trp43 and Trp56, respectively). Accordingly, vertebrate eIF4Es have been grouped into three classes: class I proteins correspond to the canonical eIF4E protein (hereafter called eIF4E1a); class II proteins, eIF4E2 or eIF4E homologous proteins (4EHP), substitute tyrosine, phenylalanine or leucine for the tryptophan residues, while the class III proteins, eIF4E3, possess a non-aromatic cysteine instead of a tryptophan in the active site [8,9]. Structures of mammalian eIF4E1a, eIF4E2 and eIF4E3 proteins resolved in NMR or crystallographic studies show the characteristic α + β domain representative of all three classes [1,2,10,11]. This domain is composed of an up to eight-stranded β-sheet that forms the cap cavity, backed by three long α-helices with a binding site for eIF4E protein partners, including eIF4G and the regulatory proteins 4E-BP and eIF4E transporter protein (4E-T).

The eIF4E-binding site in eIF4G, YXXXXLϕ (where X is any residue and ϕ is hydrophobic), is also present in 4E-BP proteins, small proteins that bind eIF4E1a when hypophosphorylated in non-proliferating cells, preventing protein synthesis. When nutrient levels are high, 4E-BP phosphorylation by mTORC1 kinase releases eIF4E1a, enabling it to interact with eIF4G. Similarly, 4E-T sequesters eIF4E1a via this YXXXXLϕ motif to reduce general translation, though when bound to mRNA, 4E-T repression is eIF4E independent [3,7,12,13]. eIF4E proteins vary in their abilities to bind the cap, eIF4G and 4E-BP [9].

Our study focuses on eIF4E1b, a class I protein, that is closely related to the canonical eIF4E1a protein. *eIF4E1b* is an evolutionary conserved gene, arising in Tetrapoda as a result of the ancestral *eIF4E* locus duplication [14]. In contrast to the ubiquitously expressed eIF4E1a protein, eIF4E1b expression is confined to ovaries, oocytes and early embryos in mice, zebrafish and *Xenopus* [14–17]. In *Xenopus* oocytes, eIF4E1b was identified as a component of the CPEB mRNP repressor complex along with the eIF4E-binding protein 4E-T, the Xp54/DDX6 RNA helicase and the RNA-binding proteins Pat and Lsm14, as well as mRNAs containing 3′ untranslated sequences recognized by CPEB [17]. Interestingly, neither recombinant nor oocyte lysate zebrafish and frog eIF4E1b proteins were able to bind immobilized m^7^GTP in contrast to their eIF4E1a counterparts, and rather than binding eIF4G, eIF4E1b interacts with 4E-T [15,17]. Inactivation of eIF4E1b in *Xenopus* oocytes by microinjection of a specific antibody or morpholino accelerated meiotic maturation of oocytes [14,17]. Moreover, the eIF4E1b-interacting partner, 4E-T, translationally repressed bound reporter mRNA. We thus proposed a repressive “closed loop” model for the silencing of CPEB target mRNAs in oogenesis involving CPEB and hence 4E-T binding to their 3′ untranslated regions and the interaction between eIF4E1b and 4E-T precluding productive binding of eIF4E1a to eIF4G [17,18].

Sequence-specific translational repression is the critical gene expression regulatory mechanism in early development where it dictates processes including oocyte maturation, establishment of embryonic axes and cell fate determination [19]. In addition to the CPEB/4E-T/eIF4E1b complex, other cases that conform to the generic model that invokes cooperation of RNA-binding proteins specifically bound to the 3′ untranslated region of mRNA with the cap-binding eIF4E-family members to form a closed loop via an intermediate protein such as 4E-T or their mutual interaction have been described. As an example of the latter, in *Drosophila*, translational control by 4EHP generates protein gradients that are essential for specifying the embryonic pattern. 4EHP is recruited to *cadual* mRNA by Bicoid that binds simultaneously to both 4EHP and the *caudal* 3′ untranslated region [4,19].

Translational regulation may be thus efficiently realized by non-canonical eIF4E proteins, even those that exhibit intrinsically low affinity for the cap structure, such as eIF4E1b or 4EHP [10,17,20]. A better insight into eIF4E-binding protein/eIF4E/mRNA complex formation will enable a more complete understanding of the mechanism of translational regulation including the participation of different eIF4E proteins.

In this study, we optimized conditions to obtain stable recombinant eIF4E1b that allowed us to investigate its cap-binding properties in relation to its canonical eIF4E1a counterpart, using both *Xenopus* and human proteins. The fluorescence assay of protein–ligand binding together with structural modeling showed conserved differences in amino acids and structural features responsible for the weaker affinity of eIF4E1b to N^7^-methyl cap analogues and, unexpectedly, an exceptionally higher specificity toward N^7^-benzyl cap analogues. Altogether, our findings provide the basis for reduced cap binding by eIF4E1b proteins, characteristic of several cap-binding proteins that mediate translational repression.

## Results

### eIF4E1a and eIF4E1b are highly related proteins, with conserved cap-binding residues, and distinguishable by several distinct amino acids

**Fig. 1:**
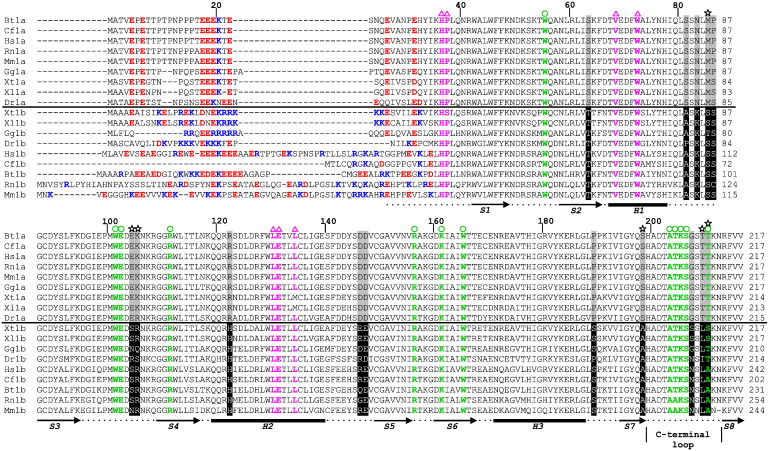
Amino acid sequence alignment of vertebrate eIF4E1a and eIF4E1b proteins of *H*. *sapiens*, *M*. *musculus*, *X*. *laevis* and *X*. *tropicalis*, *D*. *rerio*, *B*. *taurus*, *R*. *norvegicus*, *C*. *familiaris* and *G*. *gallus*, performed with CLUSTALW2. Residues in red and blue show negatively and positively charged amino acids within the N-terminus, respectively. The conserved amino acids that distinguish eIF4E1a (gray) and eIF4E1b (black) proteins are highlighted. The amino acids of the eIF4E1a cap-binding pocket and binding sites for eIF4G/4E-BP proteins are marked with circle and triangle symbols, respectively, with green and magenta shading, respectively, showing their conservation. Starred residues indicate the residues whose impact on cap binding we checked experimentally. Secondary structural elements of α-helices (*H1*–*H3*) and β-strands (*S1*–*S8*) are shown according to the crystal structure of human eIF4E1a in complex with m^7^GTP or m^7^GpppA [2].

First, we compared the sequences of eight pairs of vertebrate eIF4E1a/eIF4E1b proteins (Fig. 1). The alignment indicates the α-helical and β-sheet regions that form the core of eIF4E1a proteins and the active-site residues, both for binding the 5′ cap, as well as eIF4G and 4E-BP [1,2]. *Xenopus* and human eIF4E1a share 84% identical residues, with the main differences located at the N-terminus and a few conservative point mutations dispersed through the rest of the proteins. Importantly, all residues participating in binding the cap and eIF4G are conserved (Fig. 1).

CLUSTALW2 analysis revealed greater than 50% identity for all eIF4E1a/eIF4E1b pairs; in the case of human and frog proteins, this is 61% and 68%, respectively. Strikingly, the N-terminal sequences of eIF4E1a and eIF4E1b proteins are distinct in all examined organisms; in eIF4E1b, these vary somewhat in length and sequence but are consistently rich in basic residues (blue in Fig. 1) in contrast to the more acidic and conserved N-termini of eIF4E1a proteins (red, Fig. 1). Stretches of basic residues, such as seen in the N-terminus of *Xenopus* eIF4E1b, can promote nuclear import; however, eIF4E1b in *Xenopus* oocytes was found exclusively in the cytoplasm [17].

Similarly to eIF4E1a, eIF4E1b proteins possess the highly conserved middle and C-terminal regions corresponding to the α + β domain characteristic of eIF4E proteins. Interestingly, most of the residues that compose the cap-binding pocket are conserved in eIF4E1b (Fig. 1, highlighted with green). Moreover, the eIF4E-binding protein site on eIF4E1a, formed by residues located within H1 and H2 helices [1,2], is also largely conserved in eIF4E1b (Fig. 1, highlighted in magenta). However, closer examination of the eIF4E1a/eIF4E1b sequences revealed a set of 15 conserved and dispersed amino acid changes, which distinguish the two protein families and which are present largely in the loop regions (colored gray/black in Fig. 1).

In summary then, sequence comparison between eIF4E1a and eIF4E1b proteins confirmed the high level of sequence identity/similarity in the middle and C-terminal regions that comprise the cap-binding pocket noted previously [8,15,17] and identified conserved charge differences at their N-termini, as well as a conserved set of discrete residue changes scattered throughout the proteins.

### Homology models of *Xenopus* eIF4E1a and eIF4E1b proteins

**Fig. 2:**
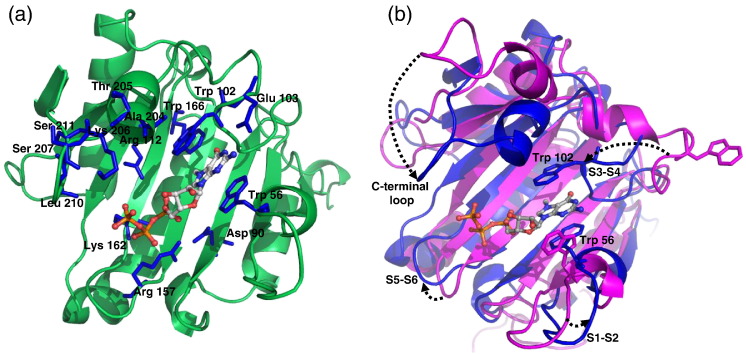
Models of the structures of *Xenopu*s eIF4E1b protein in apo and cap-bound form. The models were predicted by MODELLER using human/mouse eIF4E1a as a template (PDB IDs: 2GPQ for apo and 1IPB for cap-bound eIF4E1a). (a) eIF4E1b in complex with the m^7^GTP. The amino acids forming the cap-binding site are indicated (blue). (b) Structural superimposition of apo (magenta) and m^7^GTP-bound (blue) *Xenopus* eIF4E1b showing rearrangements of loops that compose the cap-binding site.

The high level of sequence identity of *Xenopus* proteins to human and mouse eIF4E1a, whose structures are known, allowed us to obtain reliable cap-free and cap-bound homology models. Structural models of cap-free eIF4E1b were built using human apo eIF4E1a as a template (see Materials and Methods for details) and cap-bound eIF4E1a and eIF4E1b with the mouse crystal complex of eIF4E1a–m^7^GTP as a template (Fig. 2 and Fig. S1).

The high level of sequence identity of *Xenopus* proteins to human and mouse eIF4E1a, whose structures are known, allowed us to obtain reliable cap-free and cap-bound homology models. Structural models of cap-free eIF4E1b were built using human apo eIF4E1a as a template (see Materials and Methods for details) and cap-bound eIF4E1a and eIF4E1b with the mouse crystal complex of eIF4E1a–m^7^GTP as a template (Fig. 2 and Fig. S1).

Models of XeIF4E1a and XeIF4E1b in complex with m^7^GTP indicate that Trp56, Trp102, Glu103 and Trp166 adopt the predicted spatial positions allowing for interaction with the m^7^G moiety of the cap structure (Fig. 2a and Fig. S1; here, we used XeIF4E1b numbering that is the same as for heIF4E1a; numbering of the XeIF4E1a sequence is shifted backward by 4 amino acids relative to XeIF4E1b). Polar and mostly charged amino acids including Asp90, Arg112, Arg157 and Lys162 are found in the vicinity of the phosphate chain and are able to form a hydrogen bond network as in human eIF4E1a and stabilize the XeIF4E1a and XeIF4E1b–cap complexes. The C-terminal amino acids, Ala204, Thr205, Lys206, Ser207 and Thr211, which interact with adenosine in the complex heIF4E1a–m^7^GpppA [2], are also mostly conserved in eIF4E1b except for Thr211, which stabilizes the ribose ring of adenosine. In human eIF4E1b, Thr211 is replaced by alanine, and in *Xenopus*, it is replaced by serine.

Models of XeIF4E1a and XeIF4E1b in complex with m^7^GTP indicate that Trp56, Trp102, Glu103 and Trp166 adopt the predicted spatial positions allowing for interaction with the m^7^G moiety of the cap structure (Fig. 2a and Fig. S1; here, we used XeIF4E1b numbering that is the same as for heIF4E1a; numbering of the XeIF4E1a sequence is shifted backward by 4 amino acids relative to XeIF4E1b). Polar and mostly charged amino acids including Asp90, Arg112, Arg157 and Lys162 are found in the vicinity of the phosphate chain and are able to form a hydrogen bond network as in human eIF4E1a and stabilize the XeIF4E1a and XeIF4E1b–cap complexes. The C-terminal amino acids, Ala204, Thr205, Lys206, Ser207 and Thr211, which interact with adenosine in the complex heIF4E1a–m^7^GpppA [2], are also mostly conserved in eIF4E1b except for Thr211, which stabilizes the ribose ring of adenosine. In human eIF4E1b, Thr211 is replaced by alanine, and in *Xenopus*, it is replaced by serine.

Although the model of apo XeIF4E1b adopts the globular α + β domain, similarly to human eIF4E1a, loops S3-S4 and S1-S2, containing the tryptophan residues Trp56 and Trp102, as well as the S5-S6 and C-terminal loop, are moved away from the cap-binding pocket (Fig. 2b; see Ref. [21]). In response to the presence of m^7^GTP, they undergo significant conformational changes approaching the ligand to make electrostatic contact with it.

Altogether then, sequence identity and modeled structures indicate the same protein fold in eIF4E1b as in eIF4E1a and a similarly arranged cap-binding pocket when proteins are in apo and cap-binding forms. However, previous studies using zebrafish and frog proteins showed that, while eIF4E1a binds m^7^GTP-Sepharose efficiently, the eIF4E1b counterparts only do so weakly, if at all [15,17]. We therefore used the fluorescence assay of protein–ligand binding to assess the mode of cap binding of eIF4E1b proteins in comparison with their eIF4E1a homologues. Our analysis included human and *Xenopus* proteins.

### Stabilizing the conformation of apo XeIF4E1b

The time-synchronized fluorescence titration method, widely used to determine the affinity constants of cap–eIF4E complexes [20,22–26], measures the quenching of the intrinsic fluorescence of tryptophan residues due to cap binding (Fig. S2). Knowledge of the equilibrium association constant *K*_as_ enables the calculation of the free-energy changes involved in the interaction between eIF4E and ligand, according to the standard equation Δ*G*° = − *RT*ln*K*_as_.

The time-synchronized fluorescence titration method, widely used to determine the affinity constants of cap–eIF4E complexes [20,22–26], measures the quenching of the intrinsic fluorescence of tryptophan residues due to cap binding (Fig. S2). Knowledge of the equilibrium association constant *K*_as_ enables the calculation of the free-energy changes involved in the interaction between eIF4E and ligand, according to the standard equation Δ*G*° = − *RT*ln*K*_as_.

**Fig. 3:**
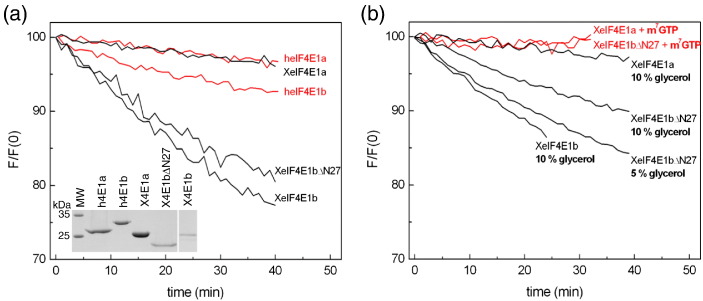
Analysis of eIF4E1a protein conformational stability. (a) Relative fluorescence intensity over time was determined for human (red) and *Xenopus* (black) eIF4E1a and eIF4E1b proteins as indicated. Insert shows the analysis of human and *Xenopus* eIF4E proteins by 15% SDS-PAGE and Coomassie Blue staining. (b) Relative fluorescence intensity over time was determined for *Xenopus* eIF4E1a and eIF4E1b in the presence of 5% or 10% glycerol in buffer as shown (black) and in the presence only of m^7^GTP (red).

The untagged eIF4E1a and eIF4E1b proteins were purified from *Escherichia coli* using the procedures previously optimized for human and mouse eIF4E1a [20]. Briefly, the proteins expressed as inclusion bodies were denatured with 6 M GdnHCl, refolded by one-step dialysis and purified to homogeneity by ion-exchange chromatography (Fig. 3a). Although all four proteins were purified from inclusion bodies with similar efficiency, the refolding step revealed their diversity of conformational stability. In particular, the refolding efficiency of *Xenopus* eIF4E1b was 5-fold lower than that of the other proteins, resulting from significant precipitation during dialysis, likely caused by its conformational instability and tendency to aggregation.

Under standard conditions used for cap-binding titration, where the protein solution is automatically stirred and the set temperature is controlled by a thermocouple, the fluorescence intensity of eIF4E proteins should be constant or decrease only slightly during the course of the experiment. This was the case for both eIF4E1a proteins, while the fluorescence signal of heIF4E1b and XeIF4E1b decreased by about 10% and 25%, respectively (Fig. 3a). Changes in fluorescence reflect mainly the rearrangements in tryptophan local environment, which can be induced by motions of protein structure [27] and/or protein aggregation. These type of changes for eIF4E proteins are abolished when bound to cap analogue that mimic their natural ligand 5′ mRNA cap (Figs. 2b and 3b), likely due to the closure and stiffening of the cap-bound protein structure [10,21]. Since we aimed to investigate the cap-binding process of different class I eIF4E proteins based on fluorescence quenching, it was essential to find conditions that stabilize the apo form of XeIF4E1b.

The 27-amino-acid N-terminal region of eIF4E1a linked to the globular α + β domain has been shown to be unstructured and not involved in binding the cap [2,21,28]. We therefore examined whether XeIF4E1b may be stabilized by removing the equivalent region, which is highly basic. First, we checked that this truncation did not alter its weak binding to cap-Sepharose (Fig. S3). We found, however, that the XeIF4E1bΔN27 protein was slightly more stable relative to the full-length protein (Fig. 3). We next tested glycerol, which is commonly used as a polar buffer component that changes the protein solvation layer and strengthens its hydrophobic core [29–31]. Indeed, buffer containing 10% glycerol provided the conditions under which the fluorescence signal of XeIF4E1bΔN27 did not decrease more than 10% (Fig. 3b). As expected, under cap-binding conditions, which stabilize protein, such an effect was no longer noticeable (Fig. 3b and Fig. S2b).

The 27-amino-acid N-terminal region of eIF4E1a linked to the globular α + β domain has been shown to be unstructured and not involved in binding the cap [2,21,28]. We therefore examined whether XeIF4E1b may be stabilized by removing the equivalent region, which is highly basic. First, we checked that this truncation did not alter its weak binding to cap-Sepharose (Fig. S3). We found, however, that the XeIF4E1bΔN27 protein was slightly more stable relative to the full-length protein (Fig. 3). We next tested glycerol, which is commonly used as a polar buffer component that changes the protein solvation layer and strengthens its hydrophobic core [29–31]. Indeed, buffer containing 10% glycerol provided the conditions under which the fluorescence signal of XeIF4E1bΔN27 did not decrease more than 10% (Fig. 3b). As expected, under cap-binding conditions, which stabilize protein, such an effect was no longer noticeable (Fig. 3b and Fig. S2b).

Using the intrinsically more stable *Xenopus* eIF4E1a protein, we then tested whether glycerol affects its cap-binding capability. Comparison of the data obtained in the presence and absence of glycerol showed that this additional buffer component reduces the association constants of eIF4E1a–cap complexes by up to 50% (Table 1, compare columns 4 and 6). We therefore report below the binding affinities of XeIF4E1a and its mutant version (named X4E1a6, see later) in buffer with and without glycerol, to compare results between human and frog eIF4E1a and eIF4E1b proteins.

### Cap-binding features of *Xenopus laevis* eIF4E1a

The purified eIF4E1a proteins were then analyzed by the time-synchronized fluorescence titration method using sets of cap analogues that differ in N^7^ and/or N^2^ guanine modification status, their phosphate chain length, the presence of a second nucleotide and modifications on ribose, all of which contribute to the binding affinity of the cap [20,22,23].

We will discuss in more detail these individual contributions below when we contrast the two families of cap-binding proteins. Overall, XeIF4E1a behaved very similarly in the cap-binding assay to human and mouse eIF4E1a proteins, determined previously [20,22]. Not surprisingly, given the high level of sequence identity between human and frog proteins (84%), they exhibited very similar cap-binding affinities (Table 1). The association constants for complexes of XeIF4E1a with various cap analogues compared to the human protein differ by only approximately 50%, and equally important, the features of binding the 5′ mRNA cap are conserved (Table 1, columns 1, 4 and 5). First, the contribution to the Gibbs free energy of binding resulting from a methyl group at N^7^ of guanine is the major factor in the cap-affinity of XeIF4E1a, as it is for heIF4E1a. Second, the cap-binding site environments of both proteins, as predicted from homology modeling (Fig. S1), react in the same manner to ethyl and benzyl substitutions at N^7^ of guanine (Tables 1 and 2). Third, the energy gain resulting from the binding of phosphate groups to the total energy of complex (Δ*G*°) is the same as for human eIF4E1a (Table 2, columns 1 and 3). Based on these experiments together with the modeled structures, we propose that, similarly to human eIF4E1a, XeIF4E1a in complex with cap is stabilized by cation–π stacking interaction of the three aromatic rings, Trp52/m^7^G/Trp98, and by a network of charged residue hydrogen bonds of the cap-binding pocket with m^7^G and the phosphate chain (Fig. S1).

We will discuss in more detail these individual contributions below when we contrast the two families of cap-binding proteins. Overall, XeIF4E1a behaved very similarly in the cap-binding assay to human and mouse eIF4E1a proteins, determined previously [20,22]. Not surprisingly, given the high level of sequence identity between human and frog proteins (84%), they exhibited very similar cap-binding affinities (Table 1). The association constants for complexes of XeIF4E1a with various cap analogues compared to the human protein differ by only approximately 50%, and equally important, the features of binding the 5′ mRNA cap are conserved (Table 1, columns 1, 4 and 5). First, the contribution to the Gibbs free energy of binding resulting from a methyl group at N^7^ of guanine is the major factor in the cap-affinity of XeIF4E1a, as it is for heIF4E1a. Second, the cap-binding site environments of both proteins, as predicted from homology modeling (Fig. S1), react in the same manner to ethyl and benzyl substitutions at N^7^ of guanine (Tables 1 and 2). Third, the energy gain resulting from the binding of phosphate groups to the total energy of complex (Δ*G*°) is the same as for human eIF4E1a (Table 2, columns 1 and 3). Based on these experiments together with the modeled structures, we propose that, similarly to human eIF4E1a, XeIF4E1a in complex with cap is stabilized by cation–π stacking interaction of the three aromatic rings, Trp52/m^7^G/Trp98, and by a network of charged residue hydrogen bonds of the cap-binding pocket with m^7^G and the phosphate chain (Fig. S1).

### eIF4E1b proteins interact distinctly with N^7^-modified guanine

As shown in Table 1, the *K*_as_ values of human and *Xenopus* eIF4E1b for m^7^GTP are approximately 3-fold lower compared with their eIF4E1a counterparts. In the case of the human proteins, these values are 68.4 ± 5.1 μM^− 1^ for eIF4E1a and 22.0 ± 1.4 μM^− 1^ for eIF4E1b. The data also show that interactions of both eIF4E1a and eIF4E1b proteins are considerably stabilized by the N^7^-methyl substitution of the cap analogue. The change in standard free energy of binding m^7^GTP by eIF4E1a (ΔΔ*G*°) compared to GTP is − 4.55 kcal/mol for human and − 4.20 kcal/mol for frog (Table 2). In the case of human eIF4E1b, the energy gain is somewhat lower and is − 2.87 kcal/mol. This difference not only is due to the weaker affinity of eIF4E1b to m^7^GTP but also reflects its approximately 5-fold higher affinity to unmethylated GTP (Table 1). For XeIF4E1bΔN27, the lower conformational stability prevented the determination of association constants for weakly binding ligands such as GTP or m^7^GMP. Bearing in mind that the phosphate chain is not as strongly stabilized in eIF4E1b as in eIF4E1a (see below), we propose that the higher binding of GTP arises from alterations in stabilizing the guanine ring in the cap-binding site.

Replacing the N^7^-methyl group with the bulkier ethyl group (et^7^GTP) decreased the association constants of both eIF4E1a proteins approximately 5-fold and of both eIF4E1b proteins 3-fold (Table 1). A distinct effect was observed for N^7^-benzyl derivative. While benzyl substitution (bn^7^GDP) did not influence eIF4E1a binding (compared with m^7^GDP), it increased the association constants of human and *Xenopus* eIF4E1b complexes with cap by approximately 5- and 2-fold, respectively. The crystal structure of human eIF4E1a with bn^7^GMP showed that the cap-binding pocket is relatively flexible and capable of conformational changes. When binding the benzyl group, the side chain of Trp102 flips about 180° exhibiting the amino group of its indole ring to the solvent, placing the benzyl ring in the hydrophobic vicinity of Trp166, His200 and Trp102 [32]. In contrast to eIF4E1a whose conformational changes did not significantly alter cap analogue complex formation, comparing ΔΔ*G*° of m^7^GDP with bn^7^GDP, in the case of eIF4E1b, this substitution resulted in considerable cap-binding enhancement, with energetic gains of ΔΔ*G*° = − 0.89 and − 0.34 kcal/mol in the case of the human and *Xenopus* protein, respectively (Table 2). These data strongly suggest that the eIF4E1b cap cavity is not as compact as in eIF4E1a since it is more able to accommodate cap analogues with ethyl and benzyl substitutions at the N^7^ position of guanine.

We also examined whether eIF4E1b could bind the hypermethylated cap structure, m^2,2,7^GTP, present in the uridine-rich small nuclear RNAs involved in splicing and present at the 5′ end of some nematode mRNAs [35]. The affinity of eIF4E1b to m^2,2,7^GTP is weak and is the same order of magnitude as for eIF4E1a, *K*_as_ ~ 0.3 μM^− 1^ (Table 1). The recently resolved structure of *Ascaris suum* eIF4E with m^7^GTP and m^2,2,7^GTP shows that the addition of two methyl groups at the N^2^ position disrupts only the hydrogen bond between the N^2^ guanine base and the carboxyl group of Glu116 (human equivalent of Glu103) from the whole network of cap–protein interactions. Unlike the methyl group at position N^7^ in the cap structure that faces inward to the core of eIF4E1a, the two methyl groups at N^2^ are exposed to the solvent [25].

Altogether, eIF4E1b binds the cap analogues approximately 3-fold less efficiently than eIF4E1a, and for all tested proteins, the methyl group at N^7^ of guanine is the major contributor to the protein's cap affinity. The enhanced binding of eIF4E1b to bn^7^GDP in contrast to eIF4E1a indicates that the base of the eIF4E1b cleft formed by the protein core is more flexible and capable of forming non-specific interactions with groups larger than methyl.

### Weaker influence of phosphate groups on stabilization of eIF4E1b–cap complexes

In addition to the positively charged 7-methylguanine moiety, the negatively charged phosphate chain is of primary importance for specific interactions of the cap with eIF4E. The α, β and γ phosphates interact with Arg112, Arg157 and Lys162 (heIF4E1a numbering) located in the S4 and S6 strands and the S5-S6 loop to stabilize the complex (Fig. 1; see Refs. [1] and [2]). Further extending the chain with δ and ε phosphate groups indicated the involvement of the C-terminal loop (S7-S8) in the stabilization process [23].

**Fig. 4:**
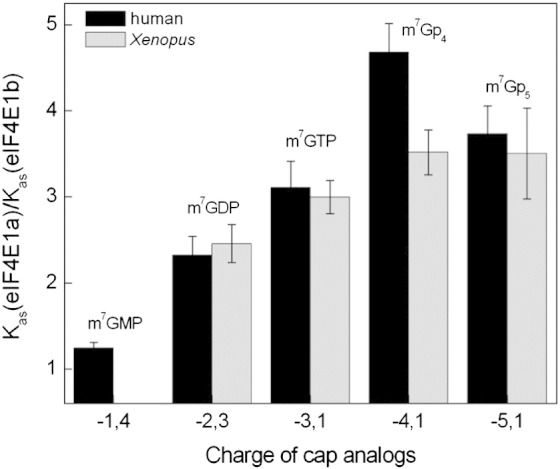
The influence of phosphate groups on the stability eIF4E1b–cap analogue complexes relative to eIF4E1a–cap complexes, described as the ratio of *K*_as_(eIF4E1a) to *K*_as_(eIF4E1b). The resultant charges of cap analogues at given pH values were estimated from experimental p*K*_a1_ values for dissociation of the N^1^ proton of N^7^-methylguanosine and from the experimental p*K*_a2_ values for the dissociation of the second proton of the terminal phosphate group [33,34].

We observed that, for human and frog eIF4E1a/eIF4E1b proteins, each added phosphate increases the stability of protein–cap binding. However, this increase of *K*_as_ values with longer phosphate chains is consistently lower in the case of both eIF4E1b proteins relative to their eIF4E1a counterparts (Fig. 4 and Table 1). For example, in the case of human proteins, the energetic gain of binding the second phosphate that makes a salt bridge with Lys162 and a hydrogen bond with Arg157 is − 1.8 kcal/mol for eIF4E1a and − 1.5 kcal/mol for eIF4E1b (Table 2), while in case of the third phosphate, which forms a hydrogen bond with Arg112, it is − 0.8 kcal/mol for eIF4E1a and − 0.6 kcal/mol for eIF4E1b and likewise for the fourth and fifth phosphates (Table 2 and Fig. 4). The reduced sensitivity of eIF4E1b proteins to the phosphate groups when binding the cap analogues may reflect the changes in amino acids in its C-terminal loop compared to eIF4E proteins.

Biophysical studies with murine eIF4E(28–217) showed that the S5-S6 loop (157–160) and the C-terminal loop (198–213), regions that close up the cap-binding pocket, have a significant influence on binding the phosphate chain. Replacement of Lys159 by neutral amino acids, such as alanine and/or introduction of negative charge in position 209 by phosphorylation of Ser or its mutation to Asp or Glu, reduced the affinity of the protein to cap analogues having two or more phosphate groups [23]. While the point mutations decreased *K*_as_ of meIF4E(28–217) to m^7^GTP 3-fold, truncation of the C-terminus [meIF4E(28–204)] reduced binding up to 5-fold [36]. Sequence alignment of vertebrate eIF4E1a/eIF4E1b proteins shows that, while there are no changes in the S5-S6 loop, the C-terminal loop distinguishes the two families (Fig. 1). Specifically, Thr210 and Thr211 are replaced with Leu and Ser(*Xenopus*)/Ala(human), respectively. Additionally, Gly in position 208 is substituted with Ser in *Xenopus* and Asn in human proteins. Given that the C-terminus significantly affects the binding of eIF4E1a with the cap phosphate chain [23,36], we propose that the presence of amino acids with longer, polar side chains instead of Gly along with the additional hydrophobic substitutions within the C-terminal loop results in the weaker stabilization of the eIF4E1b–cap complex by the phosphate chain.

### 2′-*O*- ribose methylation does not enhance eIF4E1b cap affinity

Our previous biophysical studies have revealed that mammalian eIF4E1a proteins bind all of the dinucleotide triphosphate cap analogues, m^7^GpppN (where N is any nucleotide) from 10- to 30-fold weaker than m^7^GTP, with even lower *K*_as_ values than those observed for eIF4E1a–m^7^GDP complexes [22,23]. Addition of the next nucleotides to the cap analogue structure slowly restored the *K*_as_ value to the level observed for m^7^GTP [36]. The same destabilizing effect by the second nucleotide in the cap analogue was observed for human and *Xenopus* eIF4E1b proteins (Table 2). As previously reported [22,23], the presence of the second nucleotide reduces the negative charge of the phosphate chain and destabilizes the intermolecular contacts between m^7^Gppp and eIF4E1a cap-binding residues. We suspect that the same mechanism is observed for eIF4E1b; however, the energy change resulting from the presence of the second nucleotide is slightly smaller (0.1–0.35 kcal/mol) for eIF4E1b than for eIF4E1a.

mRNA in higher eukaryotes is methylated at the 2′-*O*- ribose of the first and sometimes also the second transcribed nucleotide [37,38]. The role of these additional groups is still elusive. Our data for eIF4E1a/eIF4E1b proteins with dinucleotides (m^7^GpppN) further methylated at the first or second nucleotide on the ribose ring do not reveal any significant differences in binding compared with their unmethylated versions (Tables 1 and 2; see Refs. [22] and [39]), further indicating that neither 2′OH groups nor their methylated forms interact with eIF4E and remain solvent exposed [1,2].

### Residues distinguishing eIF4E1a and eIF4E1b–cap interactions

**Fig. 5:**
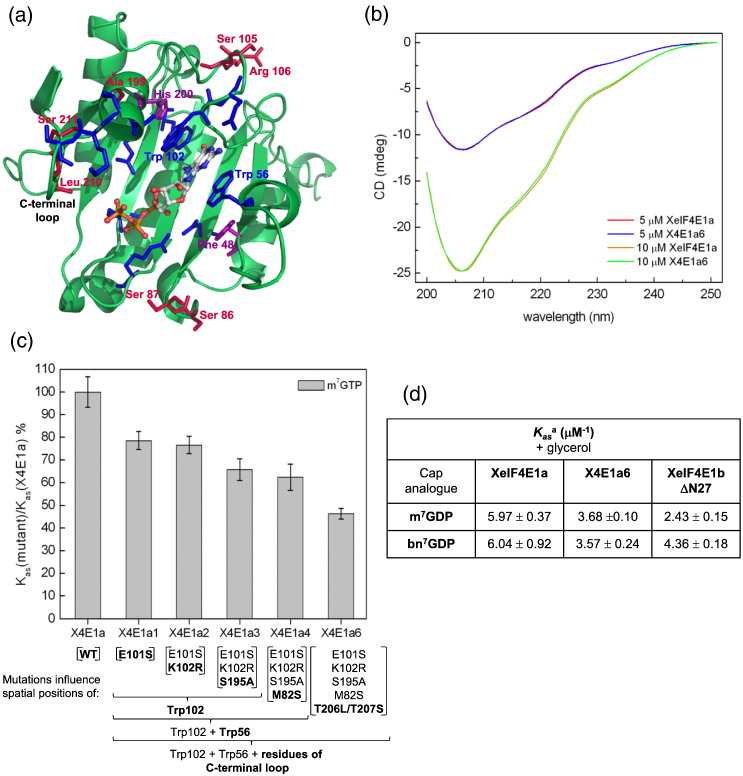
(a) Homology model of eIF4E1b in complex with m^7^GTP. The residues forming the cap-binding site are indicated (blue). Amino acids conserved in eIF4E1b and distinct to those in eIF4E1a proteins positioned in the neighborhood of the cap-binding site are highlighted in red. Residues mediating cap binding that influence positions of Trp56 and Trp102 are indicated in purple. (b) Far-UV CD spectra of XeIF4E1a and X4E1a6 performed at two protein concentrations: 5 and 10 μM. (c) Influence of mutations in XeIF4E1a on association constants. The mutations that introduce XeIF4E1b residues into XeIF4E1a are listed in brackets under the name of mutants, with the amino acids that directly interact with the cap marked below. (d) The equilibrium association constants, *K*_as_, for complexes of XeIF4E1a, its mutated form X4E1a6 and XeIF4E1b ΔN27 with m^7^GDP and bn^7^GDP.

Our analysis of vertebrate eIF4E1a and eIF4E1b sequences identified the amino acids that consistently differentiate the two groups of proteins (Fig. 1). Moreover, the three-dimensional model structure of XeIF4E1b with m^7^GTP showed that some of these residues are proximal to the amino acids that bind the cap (Fig. 5a). These include Ser86(Met), Ser87(Pro), Ser105(Glu), Arg106(Lys), Ala199(Ser), Leu210(Thr) and Ser211(Thr) (XeIF4E1b numbering, in brackets are shown the eIF4E1a amino acids). We hypothesized that these residue changes in eIF4E1b are a significant factor in its lower cap-binding affinity. The presence in eIF4E1b of Ser105 and Arg106 instead of Glu and Lys may directly influence the position of Trp102 and hence modify the stacking interaction with the cap. Additionally, Ala199 in place of Ser may induce changes in the orientation of the Trp102 indole ring by influencing the position of His200 located close to Trp102. Furthermore, cap-binding stacking interaction may be weakened by changes of the Trp56 environment, resulting from alterations in the position of Phe48 likely induced by the presence Ser86 and Ser87 instead of Met and Pro, respectively. Replacement of Thr in positions 210 and 211 by Leu and Ser is also likely to be important, as they are located in the C-terminal loop responsible for binding the phosphate chain and second cap nucleoside.

We tested the effect on cap-binding activity of these positions in the fluorescence assay with six mutated recombinant versions of *Xenopus* eIF4E1a (Fig. S4). The amino acids were introduced in XeIF4E1a sequentially in the order: E101S/K102R/S195A/M82S/T206L/T207S (numbering of the XeIF4E1a sequence is shifted backward by 4 amino acids relative to XeIF4E1b). The mutants were named with the last digit indicating the number of changed positions, for example, X4E1a2 possesses 2 mutated positions. Circular dichroism (CD) analysis indicates that these mutations do not change the secondary structure content of XeIF4E1a as the profile of far-UV CD spectra is the same for wild type and mutant protein possessing all six substitutions, X4E1a6 (Fig. 5b). The fluorescence titration data shown in Fig. 5c and Table S1 indicate that when all six residues were replaced, the association constant measured with m^7^GTP decreased by 50% compared to wild-type XeIF4E1a. The biggest contribution to the weaker cap binding of XeIF4E1a, in each case about 20%, resulted from changing Glu101 to Ser and Thr206 to Leu coupled with Thr207 to Ser, with the remaining mutations having only minor effects. Comparison of human apo eIF4E1a to the cap-bound form shows that Trp102 (Trp98 in XeIF4E1a) rotates into the cap-binding site and nearby residues also undergo conformational rearrangements [21]. Glu101 is a solvent-accessible amino acid and is proximal to Trp98 in the S3-S4 loop. Its evident effect on the binding affinity of XeIF4E1a when mutated to Ser demonstrates that properties of the S3-S4 loop amino acids influence the orientation of the Trp98 indole ring that stacks with 7-methylguanine. Attenuation of the cap-binding affinity of XeIF4E1a resulting from mutating Thr206 and Thr207 is consistent with our results mentioned above showing significant influence of C-terminal loop residues on binding phosphate and the second nucleotide.

We tested the effect on cap-binding activity of these positions in the fluorescence assay with six mutated recombinant versions of *Xenopus* eIF4E1a (Fig. S4). The amino acids were introduced in XeIF4E1a sequentially in the order: E101S/K102R/S195A/M82S/T206L/T207S (numbering of the XeIF4E1a sequence is shifted backward by 4 amino acids relative to XeIF4E1b). The mutants were named with the last digit indicating the number of changed positions, for example, X4E1a2 possesses 2 mutated positions. Circular dichroism (CD) analysis indicates that these mutations do not change the secondary structure content of XeIF4E1a as the profile of far-UV CD spectra is the same for wild type and mutant protein possessing all six substitutions, X4E1a6 (Fig. 5b). The fluorescence titration data shown in Fig. 5c and Table S1 indicate that when all six residues were replaced, the association constant measured with m^7^GTP decreased by 50% compared to wild-type XeIF4E1a. The biggest contribution to the weaker cap binding of XeIF4E1a, in each case about 20%, resulted from changing Glu101 to Ser and Thr206 to Leu coupled with Thr207 to Ser, with the remaining mutations having only minor effects. Comparison of human apo eIF4E1a to the cap-bound form shows that Trp102 (Trp98 in XeIF4E1a) rotates into the cap-binding site and nearby residues also undergo conformational rearrangements [21]. Glu101 is a solvent-accessible amino acid and is proximal to Trp98 in the S3-S4 loop. Its evident effect on the binding affinity of XeIF4E1a when mutated to Ser demonstrates that properties of the S3-S4 loop amino acids influence the orientation of the Trp98 indole ring that stacks with 7-methylguanine. Attenuation of the cap-binding affinity of XeIF4E1a resulting from mutating Thr206 and Thr207 is consistent with our results mentioned above showing significant influence of C-terminal loop residues on binding phosphate and the second nucleotide.

We also compared the cap-binding properties of X4E1a6 to those of XeIF4E1bΔN27 (Fig. 5d, Table 1 and Table S1). Though the changed residues in X4E1a6 reduced its cap affinity almost 2-fold thus resembling XeIF4E1b, however, they did not alter its sensitivity to the benzyl cap analogue. In contrast to eIF4E1b showing increased binding toward bn^7^GDP, X4E1a6 binds bn^7^GDP similarly to m^7^GDP, as did wild-type XeIF4E1a (Fig. 5d). We conclude that the introduced mutations do not impact on the core of the protein being able to accommodate the benzyl group but do influence stacking interaction with guanine and are responsible, in large part, for the reduced affinity of eIF4E1b to the cap.

We also compared the cap-binding properties of X4E1a6 to those of XeIF4E1bΔN27 (Fig. 5d, Table 1 and Table S1). Though the changed residues in X4E1a6 reduced its cap affinity almost 2-fold thus resembling XeIF4E1b, however, they did not alter its sensitivity to the benzyl cap analogue. In contrast to eIF4E1b showing increased binding toward bn^7^GDP, X4E1a6 binds bn^7^GDP similarly to m^7^GDP, as did wild-type XeIF4E1a (Fig. 5d). We conclude that the introduced mutations do not impact on the core of the protein being able to accommodate the benzyl group but do influence stacking interaction with guanine and are responsible, in large part, for the reduced affinity of eIF4E1b to the cap.

### Cap-Sepharose binding assays

**Fig. 6:**
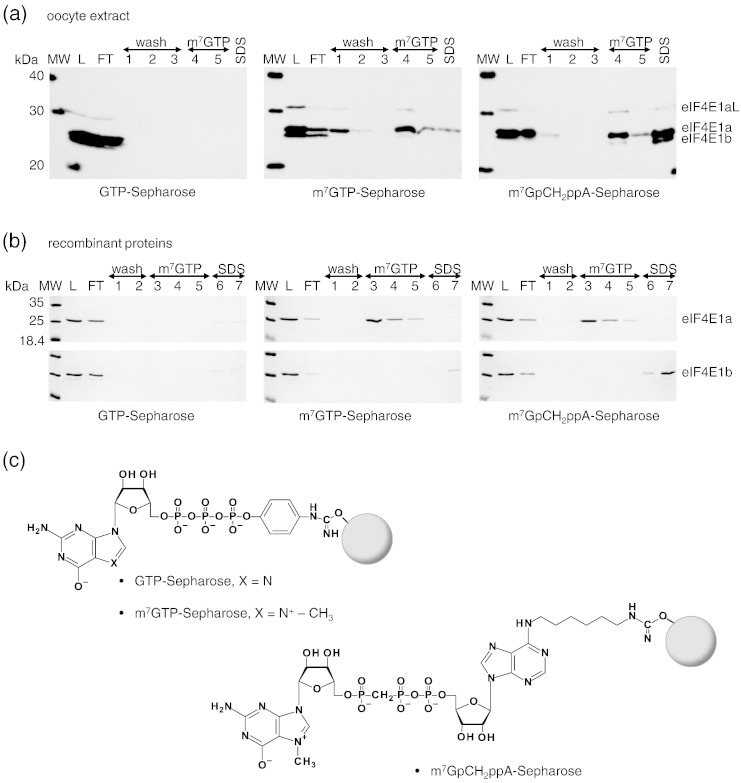
Cap-Sepharose binding assays. (a) Lysates from mid-stage *Xenopus* oocytes were analyzed by affinity chromatography with control GTP-Sepharose, m^7^GTP-Sepharose and m^7^GpCH_2_ppA-Sepharose. Aliquots of load (L), flow-through (FT), wash (lanes 1–3), m^7^GTP elution (lanes 4 and 5) and final SDS-sample buffer (SDS) fractions were analyzed by Western blotting using an-eIF4E1 antibody, which detects eIF4E1a (both an alternatively spliced isoform L, eIF4E1aL, and the canonical isoform, eIF4E1a) and eIF4E1b. (b) Analysis of binding recombinant proteins XeIF4E1a and XeIF4E1b to GTP-Sepharose, m^7^GTP-Sepharose and m^7^GpCH_2_ppA-Sepharose by 15% SDS-PAGE and Coomassie Blue staining. (c) The structures of GTP-Sepharose, m^7^GTP-Sepharose and m^7^GpCH_2_ppA-Sepharose are shown.

Previously, we and others found that eIF4E1b, whether in cell lysates or in recombinant form, bound only very weakly to cap-Sepharose, if at all [15,17]. In agreement, our quantitative assays show that, in solution, eIF4E1b binds the m^7^GTP cap analogue less well than eIF4E1a, though the effect is only 3-fold, less pronounced than predicted by these earlier studies (Table 1). However, the affinity of the immobilized cap analogue may differ significantly from its free counterpart due to the coupling step generating possible steric hindrance by close proximity of the cap to the bulky Sepharose bead [40]. To examine this possibility, we performed cap-Sepharose assays using control GTP-Sepharose, m^7^GTP-Sepharose and m^7^GpCH_2_ppA-Sepharose [40] with the use of oocyte lysate and recombinant proteins. m^7^GpCH_2_ppA-Sepharose is a modified form of m^7^GTP-Sepharose with a hexylene spacer linking the cap to the resin (Fig. 6c). Lysates were prepared from mid-stage *Xenopus* oocytes, when the levels of eIF4E1a and eIF4E1b proteins are approximately equal, as judged by Western blot analysis [17], and portions were bound to the three matrices, which were washed and bound proteins eluted with 70 μM m^7^GTP and subsequently with SDS buffer (Fig. 6a). A low concentration of GTP (0.1 mM) was included in all the buffers, to minimize non-specific binding. Fractions of load, flow-through and bound proteins were assessed by Western blotting and a pan-eIF4E1 antibody (Fig. 6a). As expected, we did not observe any oocyte protein binding to GTP-Sepharose, and in the case of m^7^GTP-Sepharose, eIF4E1a was specifically eluted from the matrix by free m^7^GTP and very much less eIF4E1b as seen before [17]. With m^7^GpCH_2_ppA-Sepharose, however, while similar amounts of eIF4E1a and eIF4E1b were eluted with m^7^GTP, SDS buffer eluted considerable amounts of eIF4E1b, as well as eIF4E1a, compared to those seen with m^7^GTP-Sepharose (Fig. 6a). In the case of recombinant proteins, equal amounts were loaded onto the Sepharose beads, and the washed and bound proteins were eluted with 100 μM m^7^GTP and then with 0.2% SDS prior to SDS-PAGE and Coomassie Blue staining. The higher concentration of free m^7^GTP compared to the oocyte assay allowed efficient elution of eIF4E1a from these matrices but any weak release of eIF4E1b with m^7^GTP was under the sensitivity level of Coomassie detection. As for the oocyte assay, SDS eluted considerable amounts of eIF4E1b from m^7^GpCH_2_ppA-Sepharose (Fig. 6b). Though the reason for the ability of SDS, and not cap analogue, to release eIF4E1b is not clear, we conclude that eIF4E1b–cap interactions can be enhanced by placing a linker between m^7^GTP or m^7^GpppN and the Sepharose bead. Since it already binds the cap better, such an enhancement is also seen with eIF4E1a to a lesser extent. The routine cap-Sepharose assay may therefore be misleading with regard to cap-binding capacities of proteins, in the absence of quantitative affinity assays, or modified cap matrices. Nevertheless, our results suggest, once again, significant differences between eIF4E1a and eIF4E1b interactions with the cap.

## Discussion

Our experimental data and homology modeling, summarized here and discussed below, demonstrate shared and distinct features of cap binding by human and *Xenopus* eIF4E1b proteins. eIF4E1a and eIF4E1b proteins are highly related, both at the sequence level and at the structural level, with conserved cap-binding residues, as are those that associate with eIF4G and 4E-BP. However, several distinct conserved amino acid changes distinguish the two families, modeled to be in the vicinity of residues that bind the cap and in the C-terminal loop. eIF4E1b binds m^7^GTP 3-fold less well than eIF4E1a, and the N^7^-methyl group is a crucial contributor to cap recognition by both proteins. However, in contrast to eIF4E1a, benzyl substitution at N^7^ guanine enhances affinity to eIF4E1b, suggesting that its core cap cavity is less compact. Another difference between the two class I families is that the complexes of eIF4E1b proteins with cap analogues are less stabilized by lengthening of the phosphate chain as seen for eIF4E1a, possibly linked to differences in certain C-terminal loop residues. Neither eIF4E1a nor eIF4E1b proteins are affected by 2′O ribose methylation, whether of first or of second cap analogue nucleotide. Interestingly, mutagenesis of several distinguishing residues in XeIF4E1a to make it resemble XeIF4E1b did indeed reduce its cap affinity by approximately 50%, verifying the influence of certain amino acids on those that bind the cap, as well as C-terminal residues. However cap binding of mutant X4E1a6 was not enhanced by benzyl at N^7^, implying that the changes did not loosen its cap cavity's compactness. Last, we showed that eIF4E1b binding to cap-Sepharose can be specifically enhanced by introducing a linker between the cap analogue and the Sepharose bead.

### eIF4E1b, cap binding and implications for the role of additional residues in cap affinity

Clearly, eIF4E1b similar to eIF4E1a shares the basic mechanism of binding the 5′ mRNA cap based on specific recognition of N^7^-methylguanine, principally mediated by two tryptophan residues and interactions with the phosphate chain by positively charged amino acids. However the 30–40% difference in sequence between eIF4E1a and eIF4E1b proteins distinguishes their affinity to the cap, as well as specificity toward selected cap analogues. This is evidenced by lower stabilization of N^7^-methylguanine and weaker binding of the phosphate chain by eIF4E1b that results in 3-fold weaker cap-binding affinity. Our studies showed that loops, S1-S2, S3-S4, S5-S6 and S7-S8, make substantial contributions to eIF4E–cap complex formation because their flexibility enables local structural changes. Binding m^7^GTP to eIF4E brings about conformational rearrangements along with residues that compose those loops and directly interact with ligand (Fig. 2b) [21]. Our sequence analysis revealed that residues that are strongly conserved in vertebrate eIF4E1a but differ from those in eIF4E1b proteins in physicochemical properties and/or size are found in those flexible regions. Mutations of six of these positions in XeIF4E1a to those found in XeIF4E1b (E101S/K102R/S195A/M82S/T206L/T207S; XeIF4E1a numbering) reduce protein binding up to 50% and make XeIF4E1a protein resemble XeIF4E1b in binding N^7^-methylmononucleotides, clearly showing that these residues modulate cap binding. Surprisingly, unlike eIF4E1a, eIF4E1b proteins show higher affinity toward benzyl derivative, even though there is no amino acid change in the hydrophobic vicinity of bn^7^G compared to eIF4E1a, suggesting further local rearrangements also in the protein's core.

The N^7^-benzyl derivatives are of interest as a potential anticancer therapeutic. In this approach, they are employed to inhibit the eIF4E1a–5′ mRNA cap interaction in oncogenic cells with increased eIF4E1a expression [41,42]. Cai *et al.* showed that benzyl or *p*-chlorobenzyl for methyl substitutions at the N^7^ position of guanine significantly increased the inhibitory potency of the monophosphates (IC_50_ values are 382 μM, 113 μM and 51 μM for m^7^GMP, bn^7^GMP and p-Cl-bn^7^GMP, respectively) but not of the diphosphates (IC_50_ are 7.50 μM and 6.76 μM for m^7^GDP and p-Cl-bn^7^GDP, respectively) [43]. The resolved structures of human eIF4E1a with bn^7^GMP or p-F-bn^7^GMP showed that both benzyl and para-fluorobenzyl groups accommodate in the hydrophobic part of the cap-binding pocket and form additional favorable protein contacts to partly compensate for loss of β and γ phosphates [32]. Indeed, later, Ghosh *et al*. revealed that bn^7^GMP binds eIF4E1a 10-fold higher than the methylated counterpart, m^7^GMP (*K*_d_ = 0.8 μM for bn^7^GMP and *K*_d_ = 7.5 μM for m^7^GMP) [44]. By contrast, in the case of the diphosphate benzyl derivative, bn^7^GDP (this study; Table 1), or the dinucleotides bn^7^GpppG and bn^7^GppppG [45], no increase in cap-binding affinity to eIF4E1a was observed. This suggests that the presence of tightly bound phosphate groups with protein could introduce some constraints for interaction of the benzyl group with eIF4E1a but not with eIF4E1b (Table 1).

Interestingly, the benzyl dinucleotide bn^7^GpppG incorporates into mRNA during *in vitro* transcription with similar efficiency as the anti-reverse cap analogue (m^7,3′O^GpppG) with a 3′O position blocked by a methyl group to force incorporation only in the correct orientation [39,45]. This result and the fact that both dinucleotide cap analogues, bn^7^GpppG and m^7,3′O^GpppG, display higher inhibitory potency and translational efficiency compared with m^7^GpppG [39,45] suggest that the benzyl for methyl substitution at N^7^ position of guanine is favorably recognized by eIF4E1a in the rabbit reticulocyte lysate system (RRL). Although there is no consistency in the influence of the benzyl substitution at N^7^ position in binding affinity to eIF4E1a (this study and Refs. [44] and [45]), all benzyl derivatives act as efficient inhibitors in model biological systems (RRL and zebrafish embryo cells) compared with their methylated counterparts [43–45]. bn^7^GMP, applied in lung, breast cancer and malignant mesothelioma cells as a membrane-permeable prodrug called 4Ei-1 [which is converted into the active compound in cells—bn^7^GMP—by the histidine triad nucleotide-binding protein 1 (Hint1)], suppresses malignant phenotypes and chemosensitizes cells to non-toxic levels of the cytotoxic drugs such as gemcitabine or pemetrexed [46,47].

Other non-canonical eIF4E proteins such as 4EHP (eIF4E2) or eIF4E3 also show lower binding to the cap structure, though the difference in association constants of these proteins for mononucleotide cap analogues relative to eIF4E1a reaches 2 orders of magnitude [10,11,20]. They share the same general fold of a central curved β-sheet flanked by three α-helices at their convex site, but unlike eIF4E1a, they differ in cap binding slot arrangements that are also modulated by loop residues. In 4EHP, stacking interactions with m^7^Gua are realized by the aromatic rings of Tyr78 and Trp124 but the length and composition of the loops in which they are located results in unfavorable ring orientation and consequently contributes to its considerably weaker cap interaction. In eIF4E3, Trp98 and Cys52 and residues of the loop preceding the small helix containing Cys52, specifically Ser43, Leu44, Pro45, Ala47 and Ala49 have direct contact with m^7^Gua. In all eIF4E proteins, contact with the phosphate chain is mediated by a network of polar and charged amino acids (eIF4E1a/1b: Asp90, Arg112, Arg157 and Lys162; eIF4E2: His110, Lys134, Arg138 and Arg174; eIF4E3: Arg152, Lys192 and Arg 84). The C-terminal loop that varies in different eIF4E subfamilies, though is quite evolutionary conserved within one, exerts considerable influence on stabilization of interactions with the phosphate chain and the second cap nucleotide and, uniquely in eIF4E3, has also contact with the ribose ring. Nevertheless, even in the context of these distinct features of cap binding by class I, II and III eIF4E proteins, they all, so far, efficiently discriminate between m^7^G and unmodified guanine [10,11,20].

Interestingly, recombinant human DcpS, a decapping enzyme, like eIF4E1b, also only binds a cap analogue well when separated by the same linker from Sepharose [40]. In this case, the binding capacity of this matrix was likely increased by the incorporation of a dinucleotide analogue, based on the structural data showing that hDcpS interacts not only with N^7^-methylguanosine but also with the second base of the cap structure [48].

### eIF4E1b, cap binding and translational control in early development

Our extensive biophysical analysis of the cap-binding properties of eIF4E1b proteins was prompted by our earlier work and those of others of the role of these proteins in translational regulation in early development (see Introduction). In these studies, cap affinity, assessed by monitoring binding of recombinant or of oocyte lysate proteins to cap-Sepharose, was estimated to be very low, as only weak if any binding was detected [15,17]. This was surprising to an extent, as, first, eIF4E1b sequences are very similar to those of eIF4E1a proteins and, second, the closed-loop model of a repressed maternal mRNA invokes eIF4E1b bound both to the cap and to an eIF4E-binding protein such as 4E-T inhibiting translation [4,7]. Moreover, 4EHP, a class II eIF4E2, considerably less related to eIF4E1a than is eIF4E1b and that binds the cap 30- to 100-fold less well than eIF4E1a [20], nevertheless performs the same functions as those proposed for eIF4E1b (see Introduction; see Ref. [49]). Weak cap binding by these proteins may allow their release from repressed mRNAs during meiotic maturation, for example, and would not inhibit general protein synthesis.

Our present study demonstrates that eIF4E1b possesses distinct features of cap binding to eIF4E, and it is pertinent to consider whether these differences, particularly our prediction that its cap cavity is more flexible, possibly reflect changes to the cap structure in oocytes and eggs. Unfortunately, it is technically difficult to assess the state of the cap on maternal mRNAs, though a study employing microinjected reporter mRNAs and cap analogues concluded that the activity of a cytoplasmic guanine-7-methyl-transferase increased substantially during oocyte maturation and stimulated translation of an injected mRNA bearing a nonmethylated GpppG cap. Moreover, the poly(A) tail and N^7^-methylation of reporter mRNA stimulated translation synergistically though independently at maturation [50]. While cytoplasmic polyadenylation of maternal mRNA has been extensively characterized and its role in translational activation is understood to a considerable extent [51,52], to our knowledge, there have been no follow-up studies to that of Gillian-Daniel *et al*. [50], and thus, the modification status of caps on maternal mRNAs remains to be established. Though previously suggested to stimulate translation at maturation [53], cap ribose methylation was reported to be very inefficient and not required for translational activation by poly(A) [50]. We found no evidence that cap ribose methylation on the first transcribed nucleotide enhanced cap binding by class I eIF4E proteins.

### eIF4E1b and eIF4E-interacting proteins

eIF4E-binding proteins dictate the physiological roles of cap-binding proteins. The principal example is eIF4G, which in complex with eIF4E mediates initiation of translation. The global mRNA inhibition of translation is promoted by 4E-BP/eIF4E1a binding, while translation of specific mRNA is negatively regulated by the following partnerships: Bicoid-4EHP [49], 4E-T-eIF4E1b [17], Cup-eIF4E [54,55], Prep1-4EHP [56] and GIGYF2-4EHP [57]. 4E-T also regulates eIF4E cellular distribution, as it can promote transport of eIF4E1a to the nucleus [58] and it recruits both eIF4E1a and eIF4E2 into processing bodies (P-bodies) [7,59–61]. In all these cases, interaction of eIF4E proteins to eIF4E-binding proteins is principally mediated through a consensus eIF4E-binding motif (YX_4_Lϕ or an extended variant YXYX_4_Lϕ), frequently located within the N-terminal portion of eIF4E partners [7,12]. Sequences downstream of the canonical binding site in eIF4G and 4E-BP may also influence interaction with eIF4E1a and may stabilize complex formation [28,62–64]. Interestingly, the downstream look-alike motif in vertebrate 4E-T, YX_4_VW, that binds eIF4E1a, eIF4E2 and eIF4E1b, contributes differentially to their 4E-T–eIF4E complex assembly [17,61]. Morita *et al*. reported that, in the case of GIGYF2 (GRB10 interacting GYF protein 2), its interaction with 4EHP is required for stabilization of both proteins in human cells [57]. One of the best-characterized eIF4E partners is *Drosophila* Cup that mediates translational repression of *oscar*, *nanos* and *gurken* mRNAs during early development [19]. Both of its eIF4E-binding sites, the canonical motif YTRSRLM and the non-canonical ELEGRLR, additively contribute to the overall conformational stability of *Drosophila* eIF4E1. Interestingly, Cup enhances almost 2-fold the cap-binding protein's affinity to m^7^GDP [65], echoing the robust increase shown by eIF4G [66]. It may be the case that 4E-T binding to eIF4E1b and eIF4E2 enhances their affinity for the cap, though this remains to be investigated. Previously, we observed that 4E-T preferentially associates with eIF4E1b rather than eIF4E1a in *Xenopus* oocytes [17,61], though the underlying reason for this is not known. It is also possible that eIF4E1b is phosphorylated or modified by ubiquitin or ubiquitin-related peptides, as shown for eIF4E2, which when modified with ISG15 binds the cap more efficiently [67,68].

Altogether, our study implicates the role of additional amino acids, located close to those that bind the cap, in modulating cap affinity, and further strengthen the evidence that C-terminal loop residues are also important for protein–cap interactions. In the future, it will be undoubtedly interesting to visualize the interactions of eIF4E1b with a capped mRNA at a structural level, ideally in the presence of the 4E-T-interacting peptide.

## Materials and Methods

### Cloning and mutagenesis

The cDNAs of *Xenopus* eIF4E1a, eIF4E1b and the N-terminal truncated form, eIF4E1bΔN27, were amplified by PCR from pGEM1 and pGEX-2T vectors [17] and cloned into the expression vector pET30a (Novagen) between NdeI and BamHI restriction sites. The human eIF4E1b gene in pUC57 vector (BIOMATIK) was similarly subcloned into pET30a vector. Point mutations in *Xenopus* eIF4E1a were introduced sequentially using the QuikChange Site-Directed Mutagenesis Kit (Stratagene) according to the manufacturer's instructions. All constructs were confirmed by sequencing. Oligonucleotides used for cloning and mutagenesis are listed in Table S2.

The cDNAs of *Xenopus* eIF4E1a, eIF4E1b and the N-terminal truncated form, eIF4E1bΔN27, were amplified by PCR from pGEM1 and pGEX-2T vectors [17] and cloned into the expression vector pET30a (Novagen) between NdeI and BamHI restriction sites. The human eIF4E1b gene in pUC57 vector (BIOMATIK) was similarly subcloned into pET30a vector. Point mutations in *Xenopus* eIF4E1a were introduced sequentially using the QuikChange Site-Directed Mutagenesis Kit (Stratagene) according to the manufacturer's instructions. All constructs were confirmed by sequencing. Oligonucleotides used for cloning and mutagenesis are listed in Table S2.

### Sequence alignment and homology modeling

Multiple sequence alignment was performed using CLUSTALW2. Protein sequences were downloaded from the National Center for Biotechnology Information database and eIF4E/4E-BP-Family Member Database [8]: *Homo sapiens* 1a: NP_001959.1, 1b: NP_001092878.1; *Mus musculus* 1a: AAH85087.1, 1b: Q3UTA9.1; *X*. *laevis* 1a: NP_001089212.1, 1b: GenBank™ BQ398016 [8]; *Xenopus tropicalis* 1a: CAJ83126.1, 1b: AAI54955.1 [8]; *Danio rerio* 1a: NP_571808.1, 1b: NP_571529.1; *Bos taurus* 1a: NP_776735.2, 1b: XP_871211.1; *Rattus norvegicus* 1a: AAH87001.1, 1b: XP_003753002.1; *Canis familiaris* 1a: XP_544992.2, 1b: XP_546215.2; *Gallus gallus* 1a: XP_420655.2, 1b: GenBank™ BX931053.2.

Comparative models of the structures of cap-free and cap-bound *X*. *laevis* eIF4E1a and eIF4E1b were obtained by the program MODELLER [69], based on the structure of human cap-free eIF4E1a (PDB ID: 2GPQ) measured in solution [21] and the crystal structure of mouse m^7^GpppG-bound eIF4E1a (the second guanosine is not visible in the structure; PDB ID: 1L8B [22]).

MODELLER generates protein structures by satisfaction of spatial restraints with simultaneous optimisation of CHARMM energies, conjugate gradients and molecular dynamics with simulated annealing. Comparative models were validated with PROCHECK [70] and WHAT_CHECK [71] that study the geometry of the structures and with VERIFY3D [72] that reports amino acid environmental problems. All protein structure figures were generated using PyMOL†

### Protein expression and purification

Human and *Xenopus* eIF4E proteins were expressed in the host strain Rosetta2(DE3) (Novagen). Culture of transformed bacteria was induced by 0.5 mM isopropyl-1-thio-β-d-galactopyranoside when the OD_600_ was ~ 1. After 3 h of incubation at 37 °C, cells were harvested and resuspended in lysis buffer [20 mM Hepes/KOH (pH 7.5), 100 mM KCl, 1 mM EDTA (*e*thylene*d*iamine*t*etraacetic *a*cid), 2 mM DTT and 10% (v/v) glycerol] and disrupted by sonication. From the centrifuged lysate (30,000*g* for 30 min), the supernatant was removed and the pellet was washed three times with wash buffer [1 M guanidine hydrochloride, 20 mM Hepes/KOH (pH 7.2), 2 mM DTT and 10% (v/v) glycerol]. The inclusion bodies were dissolved in buffer containing 6 M guanidine hydrochloride, 50 mM Hepes/KOH (pH 7.2), 10% (v/v) glycerol and 2 mM DTT, and cell debris was removed by centrifugation (43,000*g* for 30 min). The protein (diluted to a concentration lower than 0.1 mg/mL) was refolded by one-step dialysis against 50 mM Hepes/KOH (pH 7.2), 100 mM KCl, 0.5 mM EDTA and 2 mM DTT and was then purified by ion-exchange chromatography on a HiTrap SP HP column (GE Healthcare) [20]. All eIF4E proteins were eluted with a linear gradient 0.1–1 M KCl in 50 mM Hepes/KOH (pH 7.2) and were analyzed at once in the fluorescent assay without freezing. We added 10% glycerol to the *Xenopus* eIF4E1b fraction immediately after elution. Additionally for *Xenopus* eIF4E1b, which possesses more positively charged amino acids than eIF4E1a, the dialysis step was preceded by partially removing the nucleic acid from the protein fraction dissolved in 6 M guanidine hydrochloride on silica membrane (Qiagen).

Purity of the collected proteins was assessed by SDS-PAGE electrophoresis and their concentration was determined spectrophotometrically using the extinction coefficients calculated from amino acid compositions with the ProtParam tool (ExPASy Server). These are ε_280nm_ = 53,400 M^− 1^ cm^− 1^ for human eIF4E1a, ε_280nm_ = 55,460 M^− 1^ cm^− 1^ for human eIF4E1b, ε_280nm_ = 49,960 M^− 1^ cm^− 1^ for frog eIF4E1a and its mutated forms and ε_280nm_ = 51,450 M^− 1^ cm^− 1^ for eIF4E1bΔN27.

### Cap analogues and chemical agents

The cap analogues were synthesized as reported previously: m^7^GMP, m^7^GDP, m^7^GTP [73], m^7^GpppG, m^7^GpppA [74], m^7^Gp_4_, m^7^Gp_5_ [23], m^2,2,7^GTP, et^7^GTP and bn^7^GTP [75]. The cap analogue concentrations were determined according to their extinction coefficients [33,43].

All chemical agents used in measurements were spectrophotometric grade and purchased from Sigma-Aldrich or Roth.

### Spectroscopic measurements and numerical data analysis

The time-synchronized titration method, originally evolved by Niedzwiecka *et al*. [22,76], was used to determine the binding affinity of proteins for cap analogues with increasing cap concentration (ranging from 2 μM to 5 mM). For human eIF4E1a and eIF4E1b and also for *Xenopus* eIF4E1a and its mutated forms, measurements were carried out using 0.1 μM protein in 50 mM Hepes/KOH (pH 7.2), 134.5 mM KCl, 0.5 mM EDTA and 1 mM DTT. For *Xenopus* eIF4E1b(ΔN27), the concentration was 0.2 μM and glycerol was added to the buffer to 10% (v/v) final concentration. Under these buffer conditions, eIF4E fluorescence is observed in a range of 300–440 nm with a maximum near 340–350 nm and m^7^GTP fluorescence partially covers the same range, occurring at 320–440 nm with a maximum near 380 nm (Fig. S2a). The gradually increasing concentration of cap analogue quenches protein fluorescence without shifting the spectrum maximum, while at high concentrations, the fluorescence signal of free cap analogue dominates over the fluorescence from other solution components including free eIF4E and eIF4E in complex with cap (Fig. S2a). Fluorescence of measured samples was excited at 280 nm (2.5 nm bandwidth) and detected at 337 nm or 330 nm (4 nm bandwidth), thus reducing the signal originated from the free cap analogues. Measurements were carried out on a LS-55 spectrofluorometer (Perkin Elmer Co.) using freshly prepared proteins, and the fluorescence intensities were corrected for sample dilution and the inner filter effect.

The time-synchronized titration method, originally evolved by Niedzwiecka *et al*. [22,76], was used to determine the binding affinity of proteins for cap analogues with increasing cap concentration (ranging from 2 μM to 5 mM). For human eIF4E1a and eIF4E1b and also for *Xenopus* eIF4E1a and its mutated forms, measurements were carried out using 0.1 μM protein in 50 mM Hepes/KOH (pH 7.2), 134.5 mM KCl, 0.5 mM EDTA and 1 mM DTT. For *Xenopus* eIF4E1bΔN27, the concentration was 0.2 μM and glycerol was added to the buffer to 10% (v/v) final concentration. Under these buffer conditions, eIF4E fluorescence is observed in a range of 300–440 nm with a maximum near 340–350 nm and m^7^GTP fluorescence partially covers the same range, occurring at 320–440 nm with a maximum near 380 nm (Fig. S2a). The gradually increasing concentration of cap analogue quenches protein fluorescence without shifting the spectrum maximum, while at high concentrations, the fluorescence signal of free cap analogue dominates over the fluorescence from other solution components including free eIF4E and eIF4E in complex with cap (Fig. S2a). Fluorescence of measured samples was excited at 280 nm (2.5 nm bandwidth) and detected at 337 nm or 330 nm (4 nm bandwidth), thus reducing the signal originated from the free cap analogues. Measurements were carried out on a LS-55 spectrofluorometer (Perkin Elmer Co.) using freshly prepared proteins, and the fluorescence intensities were corrected for sample dilution and the inner filter effect.

Equilibrium association constants (*K*_as_) were determined by fitting the theoretical dependence of fluorescence intensity on the total concentration of the cap analogue to the experimental data points, according to the equation described previously (see Ref. [22] and Fig. S2c). In the theoretical model, we consider fluorescence of all solution components: active ([*P*^act^]) and inactive ([*P*^inact^]) fraction of protein, protein in complex with cap ([*P*^act^*L*]) and free ligand ([*L*]). Along with *K*_as_ and the concentration of the active protein [*P*_0_^act^], other parameters such as *f*_L_ and ∆*f* = *f*_Pact_ − *f*_PL_ are also gained from the fit, where *f*_L_ represents the fluorescence efficiency of free cap analogue and ∆*f* represents the difference between the fluorescence efficiencies of the apo-protein and the complex (Fig. S2c). In this model, the determined equilibrium association constant does not dependent on the excitation or the observation wavelength (Fig. S2).

Equilibrium association constants (*K*_as_) were determined by fitting the theoretical dependence of fluorescence intensity on the total concentration of the cap analogue to the experimental data points, according to the equation described previously (see Ref. [22] and Fig. S2c). In the theoretical model, we consider fluorescence of all solution components: active ([*P*^act^]) and inactive ([*P*^inact^]) fraction of protein, protein in complex with cap ([*P*^act^*L*]) and free ligand ([*L*]). Along with *K*_as_ and the concentration of the active protein [*P*_0_^act^], other parameters such as *f*_L_ and ∆*f* = *f*_Pact_ − *f*_PL_ are also gained from the fit, where *f*_L_ represents the fluorescence efficiency of free cap analogue and ∆*f* represents the difference between the fluorescence efficiencies of the apo-protein and the complex (Fig. S2c). In this model, the determined equilibrium association constant does not dependent on the excitation or the observation wavelength (Fig. S2).

The final *K*_as_ was calculated as a weighted average of 2–6 independent titrations with the weights taken as the reciprocal of the numerical standard deviation squared. Numerical, nonlinear least-squares regression analysis was performed using Origin 6.0 (Microcal Software Inc.). Obtained association constants were used to determine the Gibbs free energy according to the standard equation Δ*G*° = − *RT*ln*K*_as_.

The CD spectra were carried out on spectrometer Chirascan™ CD (Applied Photophysics) in quartz cuvette with optical length of 0.1 mm, in 50 mM phosphate buffer and 125 mM NaClO_4,_ at 20 °C.

### Cap-Sepharose binding assays

Cap-Sepharose binding assays were performed with *Xenopus* oocyte lysates, with RRL extract in which *Xenopus* eIF4E1 proteins were translated and with purified recombinant proteins. Lysates were prepared from stage III and IV oocytes as described previously [17], and aliquots in HKE buffer [50 mM Hepes (pH 7.4), 150 mM KCl, 0.5 mM EDTA, 0.5 mM ethylene glycol bis(β-aminoethyl ether) *N*,*N*′-tetraacetic acid, 0.1 mM GTP and 14 mM β-mercaptoethanol] were bound to GTP-Sepharose, m^7^GTP-Sepharose and m^7^GpCH_2_ppA-Sepharose [40]. Following binding and washing steps, we eluted bound proteins with 70 μM m^7^GTP and then with SDS buffer (approximately 1% SDS final). Aliquots of each fraction were subsequently analyzed by SDS-PAGE and Western blotting, developed with an anti-eIF4E1 rabbit antibody and ECL [17]. mRNAs encoding the full-length *Xenopus* eIF4E1a and eIF4E1b proteins, as well as a truncated version of *Xenopus* eIF4E1b missing the first 27 amino acids, were transcribed and subsequently translated *in vitro*, as described previously [17]. The rabbit reticulocyte lysates were then bound to m^7^GTP-Sepharose (Pharmacia), and following several washes, we eluted the bound proteins with GTP and then m^7^GpppG [17]. Aliquots of each fraction were analyzed by SDS-PAGE and autoradiography.

Purified recombinant proteins were bound to GTP-Sepharose, m^7^GTP-Sepharose and m^7^GpCH_2_ppA-Sepharose in 50 mM Hepes/KOH (pH 7.4), 150 mM KCl, 0.5 mM EDTA and 1 mM DTT. Following binding and washing steps, we eluted bound proteins with 100 μM m^7^GTP and then with 0.2% SDS buffer. Aliquots of each fraction were subsequently analyzed by SDS-PAGE and Coomassie Blue staining.

The following are the supplementary data related to this article.

Table S1 Equilibrium association constants, *K*_as_, for complexes of *Xenopus* eIF4E1a mutants with a series of cap analogues

Table S2 Oligonucleotide sequences used for cloning and mutagenesis

Fig. S1 Model of the structure of *Xenopus* eIF4E1a. The canonical eIF4E1a-cap binding motif formed by three tryptophans and a net of charged amino acids are shown in the model structure of *Xenopus* eIF4E1a with m^7^GTP.

Fig. S2 (a) Fluorescence spectra of apo human eIF4E1a (0.1 μM, black line) and in the presence of increasing m^7^GTP concentration (blue lines). The m^7^GTP concentration is indicated below every curve. Fluorescence spectrum of free m^7^GTP (5 μM) without protein is also introduced (red line). (b) Fluorescence titration curves for binding of human and *Xenopus* eIF4E1a and eIF4E1b proteins to m^7^GTP observed at λ_excitation_ = 280 nm and λ_observation_ = 337 nm. The interaction between eIF4E1a proteins and cap analogues results in the quenching of their intrinsic tryptophan fluorescence. The observed increasing fluorescence intensity at a higher concentration of m^7^GTP, when protein is saturated with ligand, originates from free cap analogue emission. The theoretical analysis for the fluorescence intensity as a function of ligand was performed according to Eq. (2) [shown in (c)] and the residuals of the fits are shown below. (d) Equilibrium association constants, *K*_as_, for complex of human eIF4E1a with m^7^GTP, determined at three different single wavelength: 337 nm, 345 nm and 370 nm. The measurements were performed in 50 mM Hepes/KOH (pH 7.2), 134.5 mM KCl, 0.5 mM EDTA and 1 mM DTT, at 20 °C.

Fig. S3 Both XeIF4E1b and XeIF4E1bΔN27 bind cap-Sepharose weakly. The cap-Sepharose binding assay was performed using control XeIF4E1a and XeIF4E1b ^35^SMet-labeled proteins as indicated, synthesized in rabbit reticulocyte lysate. Aliquots of load (L), flow-through (FT), wash (lanes 1–4 and 7), GTP elution (lanes 5 and 6) and m^7^GpppG elution (lanes 8 and 9) fractions were analyzed by 15% SDS-PAGE and autoradiography.

Fig. S4 (a) Electrophoretic analysis of *Xenopus* eIF4E1a mutants. Proteins were separated by 15% SDS-PAGE and visualized by Coomassie staining. (b) Table of XeIF4E1a mutant names and amino acid modifications introduced in these proteins.

Supplementary data to this article can be found online at http://dx.doi.org/10.1016/j.jmb.2014.11.009.

## Figures and Tables

**Table 1:** Equilibrium association constants, *K*_as_, for complexes of human and *Xenopus* eIF4E1a and eIF4E1b proteins with the indicated series of cap analogues

Cap analogue	*K*_as_ (μM^− 1^) − glycerol		Ratio *K*_as_ (h4E1a) to *K*_as_ (h4E1b)	*K*_as_ (μM^− 1^) − glycerol	Ratio *K*_as_ (h4E1a) to *K*_as_ (X4E1a)	*K*_as_^a^ (μM^− 1^) + glycerol		Ratio *K*_as_^a^ (X4E1a) to *K*_as_^a^ (X4E1bΔN27)
	heIF4E1a	heIF4E1b		XeIF4E1a		XeIF4E1a	XeIF4E1bΔN27	
*Mononucleotides modified at guanine ring*								
GTP	0.027 ± 0.001^b^	0.159 ± 0.040	0.17	0.031 ± 0.006	0.87	0.055 ± 0.021	—	—
m^7^GTP	68.4 ± 5.1^b^	22.0 ± 1.4	3.11	42.8 ± 2.0	1.60	23.34 ± 0.88	7.80 ± 0.41	2.99
et^7^GTP	13.04 ± 0.31	7.34 ± 0.34	1.78	7.89 ± 0.57	1.65	4.97 ± 0.22	2.26 ± 0.20	2.20
bn^7^GDP	16.79 ± 0.56	35.4 ± 1.6	0.47	10.62 ± 0.98	1.58	6.04 ± 0.92	4.36 ± 0.18	1.38
m^2,2,7^GTP	0.362 ± 0.008	0.272 ± 0.009	1.33	0.295 ± 0.021	1.22	0.176 ± 0.011	0.253 ± 0.046	0.70

*N^7^-methylated mononucleotides with increasing number of phosphate groups*								
m^7^GMP	0.78 ± 0.04^b^	0.628 ± 0.016	1.24	0.567 ± 0.012	1.37	0.256 ± 0.018	—	—
m^7^GDP	17.76 ± 0.34^b^	7.64 ± 0.71	2.32	11.42 ± 0.50	1.56	5.97 ± 0.37	2.43 ± 0.15	2.45
m^7^GTP	68.4 ± 5.1^b^	22.0 ± 1.4	3.11	42.8 ± 2.0	1.60	23.34 ± 0.88	7.80 ± 0.41	2.99
m^7^Gp_4_	419 ± 23	89.6 ± 4.0	4.68	268 ± 10	1.57	151.1 ± 8.6	43.0 ± 2.0	3.51
m^7^Gp_5_	547 ± 31	146.6 ± 9.9	3.73	378 ± 44	1.44	230 ± 31	65.6 ± 4.2	3.50

*Dinucleotides*								
m^7^GpppG	5.94 ± 0.39^b^	3.53 ± 0.11	1.68	3.98 ± 0.11	1.49	2.291 ± 0.065	0.883 ± 0.032	2.59
m^7^GpppA	3.97 ± 0.21^b^	2.158 ± 0.053	1.84	2.015 ± 0.028	1.97	1.22 ± 0.053	0.615 ± 0.042	1.99
m^7^Gpppm^2′-*O*^G	4.77 ± 0.13	3.568 ± 0.071	1.34	—	—	2.189 ± 0.026	—	—
m^7,2′-*O*^GpppG	6.13 ± 0.34^b^	4.63 ± 0.12	1.32	—	—	1.96 ± 0.24	—	—

a Measurements were carried out in 10% glycerol whose presence in the buffer decreases the cap affinity to protein almost 2-fold.

b Data were taken from Ref. [20].

**Table 2:** Changes in the standard Gibbs free energy, ΔΔ*G*^o^, showing the contribution of structural elements of the 5′ mRNA cap to the binding free energy of eIF4E–cap complexes

	ΔΔ*G*^o^ (kcal/mol) − glycerol			ΔΔ*G^o^*^a^ (kcal/mol) + glycerol	
	heIF4E1a	heIF4E1b	XeIF4E1a	XeIF4E1a	XeIF4E1bΔN27
*Modifications on the guanine ring*					
GTP → m^7^GTP	− 4.555 ± 0.050	− 2.87 ± 0.15	− 4.20 ± 0.12	− 3.52 ± 0.23	—
GTP → et^7^GTP	− 3.590 ± 0.028	− 2.23 ± 0.15	− 3.21 ± 0.13	− 2.62 ± 0.23	—
m^7^GTP → et^7^GTP	0.965 ± 0.045	0.639 ± 0.045	0.984 ± 0.050	0.900 ± 0.035	0.722 ± 0.059
m^7^GDP → bn^7^GDP	0.033 ± 0.022	− 0.893 ± 0.060	0.042 ± 0.059	− 0.006 ± 0.096	− 0.341 ± 0.044
m^7^GTP → m^2,2,7^GTP	3.052 ± 0.045	2.557 ± 0.041	2.897 ± 0.051	2.844 ± 0.042	2.00 ± 0.11

*Elongation of the phosphate chain*					
m^7^GMP → m^7^GDP	− 1.820 ± 0.028	− 1.455 ± 0.056	− 1.748 ± 0.028	− 1.832 ± 0.054	—
m^7^GDP → m^7^GTP	− 0.785 ± 0.045	− 0.616 ± 0.065	− 0.769 ± 0.037	− 0.794 ± 0.043	− 0.679 ± 0.048
m^7^GTP → m^7^Gp_4_	− 1.055 ± 0.054	− 0.817 ± 0.044	− 1.067 ± 0.035	− 1.087 ± 0.040	− 0.994 ± 0.041
m^7^Gp_4_ → m^7^Gp_5_	− 0.155 ± 0.046	− 0.287 ± 0.047	− 0.202 ± 0.071	− 0.244 ± 0.086	− 0.246 ± 0.046

*Addition of the second nucleotide and methylation of the ribose ring*					
m^7^GTP → m^7^Gp_3_G	1.423 ± 0.058	1.065 ± 0.040	1.382 ± 0.032	1.351 ± 0.028	1.268 ± 0.037
m^7^GTP → m^7^Gp_3_A	1.658 ± 0.053	1.352 ± 0.039	1.779 ± 0.029	1.717 ± 0.033	1.479 ± 0.050
m^7^Gp_3_G → m^7,2′-*O*^Gp_3_G	− 0.018 ± 0.050	− 0.157 ± 0.022	—	0.091 ± 0.074	—
m^7^Gp_3_G → m^7^Gp_3_m^2′-*O*^G	0.128 ± 0.041	− 0.006 ± 0.021	—	0.026 ± 0.018	—

a Refers to measurements carried out in 10% glycerol.

## Footnotes

† http://www.pymol.org/.
