# Supplementary material for: Distinct Features of Cap Binding by eIF4E1b Proteins
Source: J Mol Biol. 2015 Jan 30;427(2):387–405. doi: 10.1016/j.jmb.2014.11.009 (PMC4306533; doi:10.1016/j.jmb.2014.11.009)
Supplement: Table S1 — Equilibrium association constants, Kas, for complexes of Xenopus eIF4E1a mutants with a series of cap analogues [file mmc1.doc]

|  | *K_as_* (μM^-1^) | | | | | |
| --- | --- | --- | --- | --- | --- | --- |
|  | X4E1a1 | X4E1a1’ | X4E1a2 | X4E1a3 | X4E1a4 | X4E1a6 |
| Positions mutated in eIF4E1a | (E101S) | (K102R) | (E101S/K102R) | (E101S/K102R/  S195A) | (E101S/K102R/  S195A/M82S) | (E101S/K102R/  S195A/M82S/  T206L/T207S) |
| m^7^GDP | 10.1 ± 1.2 | 11.09 ± 0.21 | 8.80 ± 0.16 | 8.53 ± 0.36 | 7.68 ± 0.40 | 6.82 ± 0.17 |
| m^7^GTP | 33.58 ± 0.66 | 34.3 ± 1.7 | 32.76 ± 0.51 | 28.1 ± 1.5 | 26.7 ± 2.2 | 19.8 ± 0.4 |
| m^7^Gp_4_ | 218 ± 39 | 232.5 ± 8.3 | 202.5 ± 6.7 | 170 ± 24 | 206 ± 12 | 143 ± 8 |
| m^7^Gp_5_ | 279 ± 34 | 382 ±16 | 280 ± 11 | - | - | 190 ± 6 |
| m^2,2,7^GTP | 0.350 ± 0.015 | 0.268 ± 0.012 | 0.306 ± 0.046 | 0.193 ± 0.019 | 0.317 ± 0.054 | 0.185 ± 0.012 |
| bn^7^GDP | 9.01 ± 0.21 | 11.22 ± 0.65 | 8.86 ± 0.16 | 9.08 ± 0.33 | 11.66 ± 0.64 | 7.09 ± 0.18 |
| et^7^GTP | 7.14 ± 0.63 | 7.38 ± 0.18 | 7.132 ± 0.088 | 6.23 ± 0.17 | 8.21 ± 0.51 | 4.41 ± 0.16 |
| m^7^GpppG | 3.38 ± 0.16 | 3.341 ± 0.090 | 2.81 ± 0.10 | 2.417 ± 0.051 | 2.87 ± 0.11 | 2.54 ± 0.15 |
| m^7^GpppA | - | - | - | 1.57 ± 0.21 | 1.48 ± 0.11 | 1.33 ± 0.11 |

Table S1
