# Supplementary material for: Distinct Features of Cap Binding by eIF4E1b Proteins
Source: J Mol Biol. 2015 Jan 30;427(2):387–405. doi: 10.1016/j.jmb.2014.11.009 (PMC4306533; doi:10.1016/j.jmb.2014.11.009)
Supplement: Table S2 — Oligonucleotide sequences used for cloning and mutagenesis [file mmc2.doc]

Table S2.

| Primer name | Vector | Sequences 5’ – 3’ |
| --- | --- | --- |
| *Xenopus* eIF4E |  |  |
| 4ENdeIFor  4EBamHIRev | pET30a | ACATGACATATGGCGGCTGTGGAACCG  GGCGAGGGATCCTTAAACAACAAATCTATTTTTAGTGG |
| 4E1bNdeIFor  4E1bBamHIRev |  | GTCACACATATGGCAGCAGCTGAAGCA  AGTAAGGGATCCTCAGACCACAAACTTGTTCTTGG |
| 4E1bΔNdeIFor  4E1bΔBamHIRev |  | CGACCGCATATGTGTGCAATTCTGGAA  AGTAAGGGATCCTCAGACCACAAACTTGTTCTTGG |
| 4EForE101S  4ERevE101S |  | G CCT ATG TGG GAA GAT AGC AAG AAC AAG CGT GG  CC ACG CTT GTT CTT GCT ATC TTC CCA CAT AGG C |
| 4EForK102R  4ERevK102R |  | CTATGTGGGAAGATGAACGCAACAAGCGTGGAGGTAG  CTACCTCCACGCTTGTTGCGTTCATCTTCCCACATAG |
| 4EForE101SK102R  4ERevE101SK102R |  | GAGCCTATGTGGGAAGATAGCCGCAACAAGCGTGGAGGTAG  CTACCTCCACGCTTGTTGCGGCTATCTTCCCACATAGGCTC |
| 4EForM82S  4ERevM82S |  | CCAATTGTCTAGTAATTTAAGCTCAGGATGTGACTACTC  GAGTAGTCACATCCTGAGCTTAAATTACTAGACAATTGG |
| 4EForS195A  4ERevS195A |  | GTGATTGGTTTTCAGGCGCATGCAGACACAGC  GCTGTGTCTGCATGCGCCTGAAAACCAATCAC |
| 4EForT206LT207S  4ERevT206LT207S |  | GCTACTAAGAGCGGCTCCCTGAGCAAAAATAGATTTGTTG  CAACAAATCTATTTTTGCTCAGGGAGCCGCTCTTAGTAGC |
| human eIF4E |  |  |
| h4E1bNdeIFor  h4E1bBamHIRev | pET30a | GTCGCACATATGCTTGCTGTTGAGGTGA  GTCACGGGATCCTCACACCACAAACTTG |
