# Supplementary figures and images for: Distinct Features of Cap Binding by eIF4E1b Proteins
Source: J Mol Biol. 2015 Jan 30;427(2):387–405. doi: 10.1016/j.jmb.2014.11.009 (PMC4306533; doi:10.1016/j.jmb.2014.11.009)

## Slide 1
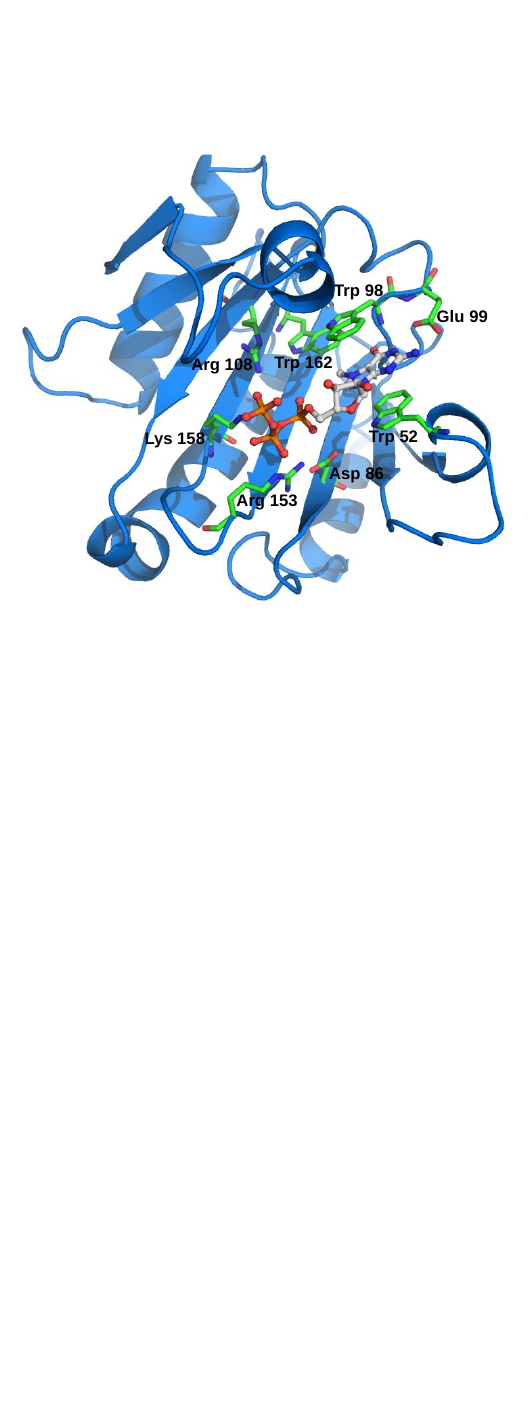

Trp 98
Glu 99
Trp 162
Arg 108
Trp 52
Lys 158
Asp 86
Arg 153

Supplement: Fig. S1 — Model of the structure of Xenopus eIF4E1a. The canonical eIF4E1a-cap binding motif formed by three tryptophans and a net of charged amino acids are shown in the model structure of Xenopus eIF4E1a with m7GTP. [file mmc3.ppt]

## Slide 1
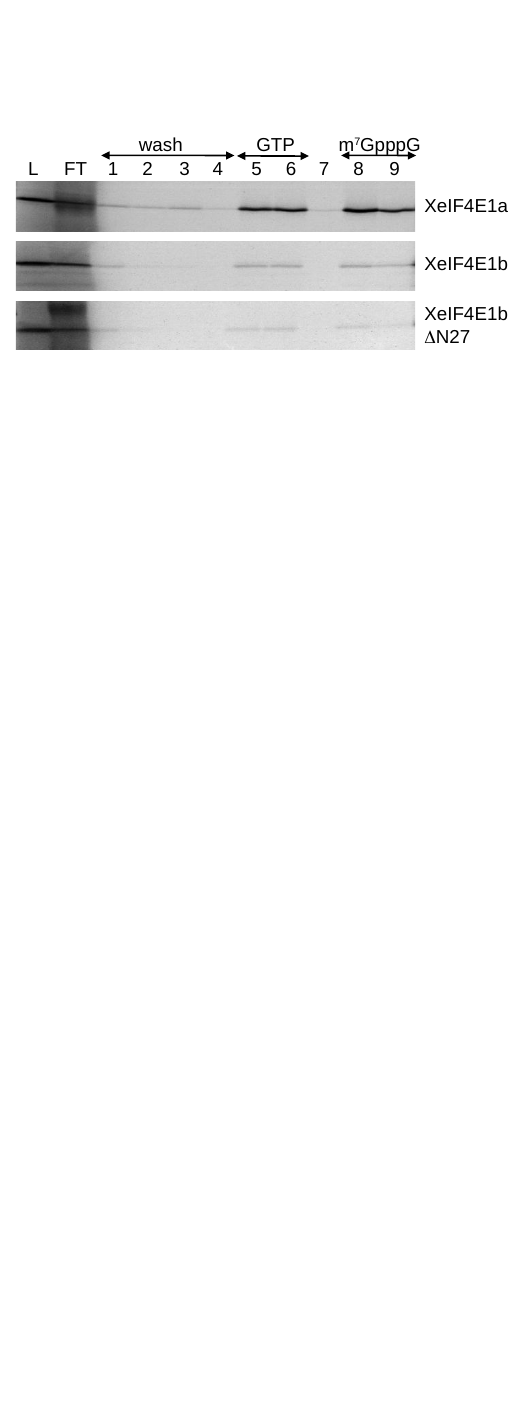

wash
GTP
m7GpppG
L
FT
1
2
3
4
5
6
7
8
9
XeIF4E1a
XeIF4E1b
XeIF4E1b
ΔN27

Supplement: Fig. S3 — Both XeIF4E1b and XeIF4E1bΔN27 bind cap-Sepharose weakly. The cap-Sepharose binding assay was performed using control XeIF4E1a and XeIF4E1b 35SMet-labeled proteins as indicated, synthesized in rabbit reticulocyte lysate. Aliquots of load (L), flow-through (FT), wash (lanes 1–4 and 7), GTP elution (lanes 5 and 6) and m7GpppG elution (lanes 8 and 9) fractions were analyzed by 15% SDS-PAGE and autoradiography. [file mmc5.ppt]
