# Supplementary material for: Distinct Features of Cap Binding by eIF4E1b Proteins
Source: J Mol Biol. 2015 Jan 30;427(2):387–405. doi: 10.1016/j.jmb.2014.11.009 (PMC4306533; doi:10.1016/j.jmb.2014.11.009)
Supplement: Fig. S2 — (a) Fluorescence spectra of apo human eIF4E1a (0.1 μM, black line) and in the presence of increasing m7GTP concentration (blue lines). The m7GTP concentration is indicated below every curve. Fluorescence spectrum of free m7GTP (5 μM) without protein is also introduced (red line). (b) Fluorescence titration curves for binding of human and Xenopus eIF4E1a and eIF4E1b proteins to m7GTP observed at λexcitation = 280 nm and λobservation = 337 nm. The interaction between eIF4E1a proteins and cap analogues results in the quenching of their intrinsic tryptophan fluorescence. The observed increasing fluorescence intensity at a higher concentration of m7GTP, when protein is saturated with ligand, originates from free cap analogue emission. The theoretical analysis for the fluorescence intensity as a function of ligand was performed according to Eq. (2) [shown in (c)] and the residuals of the fits are shown below. (d) Equilibrium association constants, Kas, for complex of human eIF4E1a with m7GTP, determined at three different single wavelength: 337 nm, 345 nm and 370 nm. The measurements were performed in 50 mM Hepes/KOH (pH 7.2), 134.5 mM KCl, 0.5 mM EDTA and 1 mM DTT, at 20 °C. [file mmc4.ppt]

## Slide 1
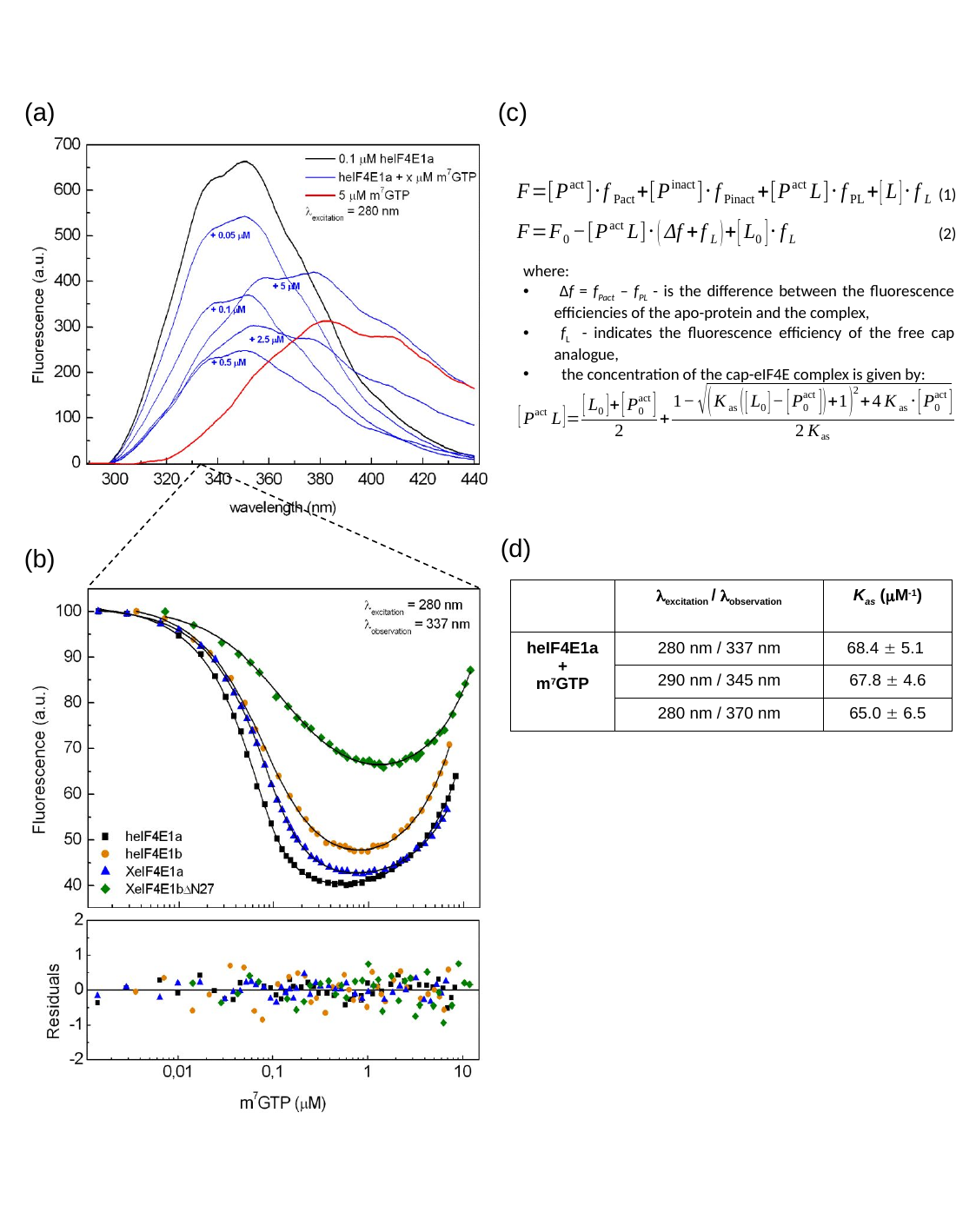

(a)
(c)
(1)
(2)
where:
 Δf = fPact – fPL - is the difference between the fluorescence efficiencies of the apo-protein and the complex,
 fL - indicates the fluorescence efficiency of the free cap analogue,
 the concentration of the cap-eIF4E complex is given by:
(d)
(b)
| | excitation / observation | Kas (μM-1) |
| --- | --- | --- |
| heIF4E1a + m7GTP | 280 nm / 337 nm | 68.4 ± 5.1 |
| | 290 nm / 345 nm | 67.8 ± 4.6 |
| | 280 nm / 370 nm | 65.0 ± 6.5 |
