# Supplementary material for: Distinct Features of Cap Binding by eIF4E1b Proteins
Source: J Mol Biol. 2015 Jan 30;427(2):387–405. doi: 10.1016/j.jmb.2014.11.009 (PMC4306533; doi:10.1016/j.jmb.2014.11.009)
Supplement: Fig. S4 — (a) Electrophoretic analysis of Xenopus eIF4E1a mutants. Proteins were separated by 15% SDS-PAGE and visualized by Coomassie staining. (b) Table of XeIF4E1a mutant names and amino acid modifications introduced in these proteins. [file mmc6.ppt]

## Slide 1
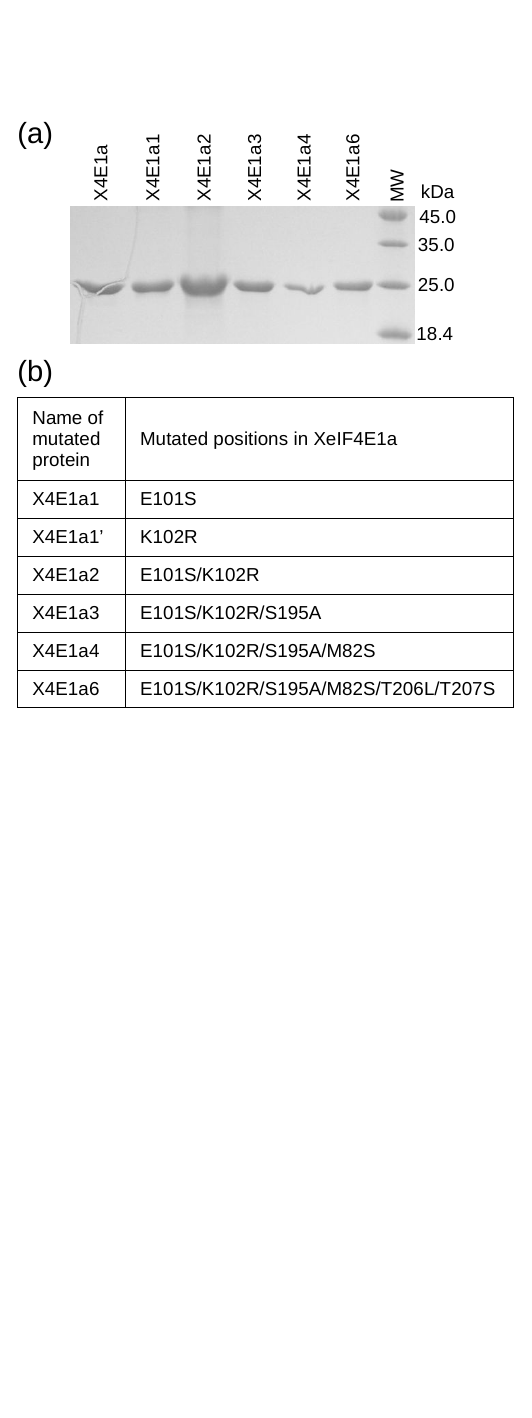

(a)
X4E1a1
X4E1a2
X4E1a3
X4E1a4
X4E1a6
X4E1a
MW
kDa
45.0
35.0
25.0
18.4
(b)
| Name of mutated protein | Mutated positions in XeIF4E1a |
| --- | --- |
| X4E1a1 | E101S |
| X4E1a1’ | K102R |
| X4E1a2 | E101S/K102R |
| X4E1a3 | E101S/K102R/S195A |
| X4E1a4 | E101S/K102R/S195A/M82S |
| X4E1a6 | E101S/K102R/S195A/M82S/T206L/T207S |
